# OGA is associated with deglycosylation of NONO and the KU complex during DNA damage repair

**DOI:** 10.1038/s41419-021-03910-6

**Published:** 2021-06-16

**Authors:** Yaqi Cui, Rong Xie, Xuefang Zhang, Yi Liu, Yixuan Hu, Yuan Li, Xiuhua Liu, Xiaochun Yu, Chen Wu

**Affiliations:** 1 School of Life Sciences, Institute of Life Sciences and Green Development, Hebei University, Baoding, Hebei Province 071002 China; 2 Department of Gastroenterology, Peking University Third Hospital, Beijing, 100191 China; 3 Westlake Laboratory of Life Sciences and Biomedicine, Hangzhou, Zhejiang China; 4 School of Life Sciences, Westlake University, Hangzhou, Zhejiang China; 5 Institute of Biology, Westlake Institute for Advanced Study, Hangzhou, Zhejiang Province China

**Keywords:** DNA damage and repair, Glycosylation

## Abstract

Accumulated evidence shows that OGT-mediated O-GlcNAcylation plays an important role in response to DNA damage repair. However, it is unclear if the “eraser” O-GlcNAcase (OGA) participates in this cellular process. Here, we examined the molecular mechanisms and biological functions of OGA in DNA damage repair, and found that OGA was recruited to the sites of DNA damage and mediated deglycosylation following DNA damage. The recruitment of OGA to DNA lesions is mediated by O-GlcNAcylation events. Moreover, we have dissected OGA using deletion mutants and found that C-terminal truncated OGA including the pseudo HAT domain was required for the recruitment of OGA to DNA lesions. Using unbiased protein affinity purification, we found that the pseudo HAT domain was associated with DNA repair factors including NONO and the Ku70/80 complex. Following DNA damage, both NONO and the Ku70/80 complex were O-GlcNAcylated by OGT. The pseudo HAT domain was required to recognize NONO and the Ku70/80 complex for their deglycosylation. Suppression of the deglycosylation prolonged the retention of NONO at DNA lesions and delayed NONO degradation on the chromatin, which impaired non-homologus end joining (NHEJ). Collectively, our study reveals that OGA-mediated deglycosylation plays a key role in DNA damage repair.

## Introduction

Posttranslational modifications play key roles in DNA damage repair, particularly in DNA double-strand break (DSB) repair [1–4]. Following DSBs, a set of posttranslational modifications, such as phosphorylation, ubiquitination, and ADP-ribosylation are induced, which regulate many biological events during DNA damage repair [5–8]. Recently, it has been shown that like other modifications, protein O-GlcNAcylation can be induced by DNA damage [9–11].

O-GlcNAcylation is catalyzed by O-GlcNAc transferase (OGT) [12, 13]. Using UDP-GlcNAc as a donor, OGT removes UDP from UDP-GlcNAc and covalently links GlcNAc residues to the side chains of serine or threonine residues in the target proteins via O-linked glycosidic bonds [14, 15]. In response to DNA damage, OGT relocates to DNA lesions and catalyzes O-GlcNAcylation at DNA lesions [9]. To date, several DNA damage-induced O-GlcNAcylation substrates have been revealed including H2AX, MDC1, DNA Pol η etc. [9, 10]. These O-GlcNAcylation events regulate DNA damage response via crosstalk with other posttranslational modifications such as phosphorylation and ubiquitination [16–19]. Therefore, it is likely that O-GlcNAcylation provides another layer of regulation in DNA damage repair.

In addition to OGT, the level of protein O-GlcNAcylation is also regulated by O-GlcNAcase (OGA) that hydrolyzes the glycosidic bond and releases GlcNAc from respective proteins [20, 21]. Human OGA (hOGA) is a 916-residue nucleocytoplasmic protein that consists of two unique functional domains including an N-terminal catalytic domain that is responsible for the removal of O-GlcNAc, and a C-terminal pseudo–histone acetyltransferase (HAT) domain (residues 707–916) that possesses sequence homology to HAT but lacks the key residues for the binding of acetyl-coenzyme A (acetyl-CoA) [22–26]. Thus, it may not have acetyltransferase activity. However, the pseudo HAT domain is evolutionarily conserved, indicating that this pseudo HAT domain may play an important role in the deglycosylation-associated functions [24, 25, 27]. Between the N-terminal catalytic domain and the C-terminal HAT domain, there is a hinge region containing several alpha helixes also named as the stalk domain [28]. Since this hinge region is not very conserved among different species, it may be a flexible region that facilitates the folding of the whole protein.

Since OGA is the solo enzyme for the removal of O-GlcNAcylation, it is very likely to participate in many O-GlcNAcylation-mediated events, such as DNA damage repair. However, to date, the role of OGA in DNA damage repair remains elusive. Here, we show that OGA relocates to the sites of DNA damage and the pseudo HAT domain of OGA plays a key role for the recruitment of OGA to DNA lesions and mediating the substrates recognition at DNA lesions. We identified DNA damage repair factors the Ku70/80 complex and NONO as substrates of OGA. Suppression of the enzymatic activity of OGA prolongs O-GlcNAcylation at DNA lesions, which in turn delays NONO degradation at DNA damage sites and impairs non-homologus end joining (NHEJ) repair pathway. Taken together, these results suggest that OGA-mediated deglycosylation promotes DNA damage repair.

## Results

### OGA mediates deglycosylation during DNA damage response

Previous studies show that O-GlcNAcylation is induced following DNA damage [9]. Since O-GlcNAcylation is a reversible posttranslational modification, we measured the kinetics of O-GlcNAcylation after DNA damage. We treated U2OS cells with 10 Gy of IR to induce DNA damage, and found that O-GlcNAcylation reached a peak level at around 10 min following IR treatment and gradually reduced to the basal level in dot blotting assays (Supplemental Fig. S1A). Although O-GlcNAcylation occurs relatively quickly in response to DNA damage, it is slower compared to DNA damage-induced PARylation. Since OGA mediates deglycosylation, we ask if OGA regulates O-GlcNAcylation following DNA damage. We treated U2OS cells with OGA inhibitor Thiamet-G (TMG) to suppress the enzymatic activity of OGA, and found that TMG treatment increased overall levels of O-GlcNAcylation (Supplemental Fig. S1B). Using additional cell line MCF10A to perform dot blotting, we obtained similar results (Supplemental Fig. S2A, B). Moreover, we used siRNA to knockdown OGA (Supplemental Fig. S3), and found that similar to the TMG treatment, the removal of DNA damage-induced O-GlcNAcylation was delayed (Supplemental Fig. S1B, C).

To investigate the role of OGA in the DNA damage response, we examined the localization of OGA in laser microirradiation assays. Following laser treatment, OGA relocated to DNA lesions within 10 min (Supplemental Fig. S1D). In addition, we found that endogeneous OGA was also recruited to DNA damage sites (Supplemental Fig. S4). When we pretreated cells with OSMI-1, an O-GlcNAcylation inhibitor, we found that the relocation of OGA was suppressed, suggesting that OGA recognized O-GlcNAcylation substrates at DNA lesions (Supplemental Fig. S1C). Collectively, these results suggest that OGA mediates deglycosylation at DNA lesions.

### The HAT domain of OGA regulates deglycosylation

**Fig. 1:**
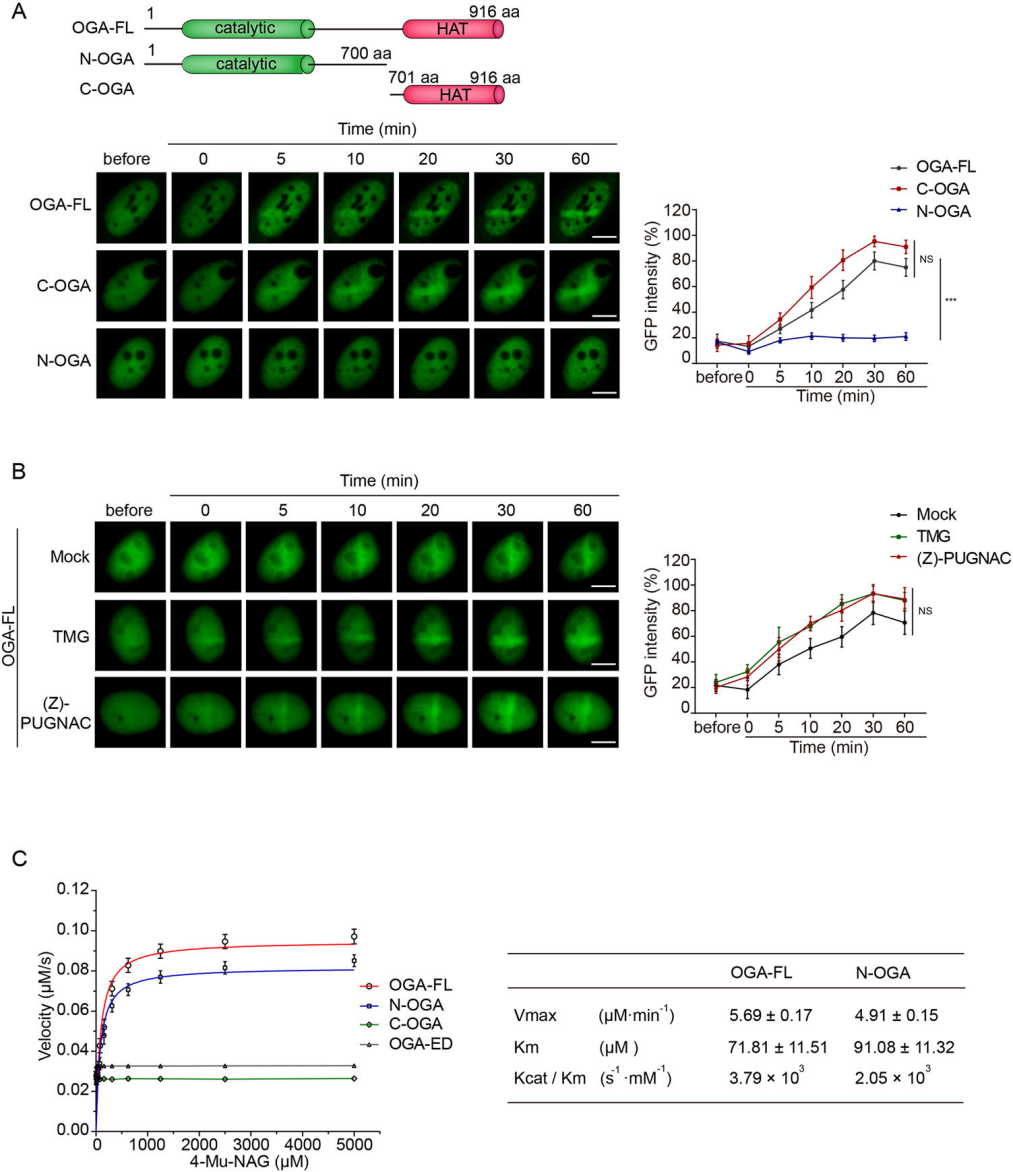
The nuclear localization of OGA in response to laser microirradiation. **A** The HAT domain plays a key role for the OGA recruitment to DNA lesions. Upper panel: diagrams showing the OGA deletion mutants. Lower panel: The full-length OGA, C-OGA, or N-OGA were expressed in U2OS cells. The cells were treated with laser microirradiation. The recruitment kinetics of full-length OGA, C-OGA, and N-OGA to laser strips were examined by measuring the peak fluorescence density of OGA at the laser strips at indicated time points using Image J (right panel). Scale bar: 5 μm. **B** Suppression of OGA’s enzymatic activities does not affect the recruitment of OGA. U2OS expressing full-length OGA were treated with OGA inhibitors ((Z)-PUGNAC (50 μM) or TMG (10 μM) for 24 h and then were subjected to laser microirradiation. The peak fluorescence density of OGA at the laser strips was quantified at indicated time points by Image J (right panel). Scale bar: 5 μm. **C** Michaelis–Menten kinetics of full-length OGA (OGA-FL), N-OGA, C-OGA, and OGA-ED. 4-Mu-NAG was incubated with recombinant OGA or its truncation mutants in the in vitro deglycosylation assays. The enzymatic parameters are: OGA-FL, Vmax = 5.69 ± 0.17 μM min^−1^, Km = 71.81 ± 11.51 μM and Kcat/Km = 3.79 × 10^3^ (s^−1^ mM^−1^); N-OGA, Vmax = 4.91 ± 0.15 μM min^−1^, Km = 91.08 ± 11.32 μM and Kcat/Km = 2.05 × 10^3^ (s^−1^ mM^−1^). Data were fitted using GraphPad Prism. Error bars represent ± SEM values derived from three independent experiments.

Since OGA has two prominent domains, we asked which domain-mediated the relocation of OGA to DNA lesions. We generated two truncated OGA mutants by deleting either the N-terminal catalytic domain or the C-terminal HAT domain (C-OGA and N-OGA). Interesting, only C-OGA but not N-OGA could relocate to DNA lesions (Fig. 1A), suggesting that the HAT domain of OGA mediates the recruitment. Moreover, we treated cells with TMG or (Z)-PUGNAC, two different OGA inhibitors, to block the catalytic pocket of OGA, OGA was still able to be recruited to DNA lesions (Fig. 1B). Taken together, these results suggest that the HAT domain but not the catalytic domain is required for the recruitment.

Next, we asked if the HAT domain regulated the enzymatic activity of OGA. We generated recombinant full-length OGA, N-OGA, and C-OGA. Using 4-Mu-NAG as a substrate, we measured enzymatic parameters of full-length OGA and the mutants including Km, Vmax, and Kcat/Km (Fig. 1C). Compared to the full-length OGA, N-OGA still retained the enzymatic activity. In contrast, the C-OGA and the enzymatically dead mutant of OGA (OGA-ED, D174A/D175A double mutations) were not able to digest the substrate (Fig. 1C). It suggests that the HAT domain itself does not catalyze deglycosylation.

### The HAT domain plays an important role in DNA damage repair

**Fig. 2:**
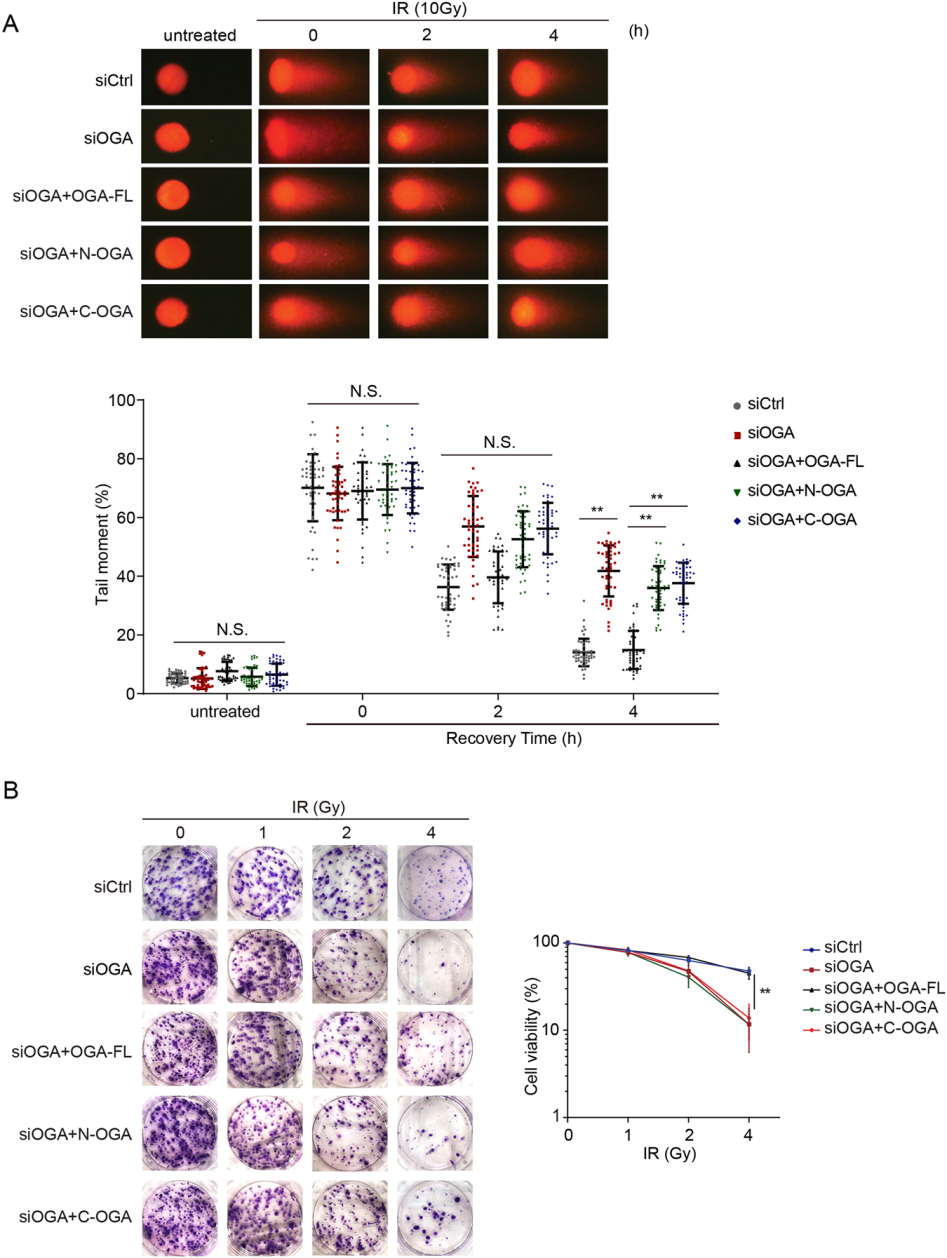
OGA participates in DNA damage repair. **A** Loss of OGA impairs DNA damage repair. Cells were treated with siOGA and were reconstituted with full-length OGA, N-OGA, or C-OGA. The cells were then treated with 10 Gy of IR. DSB repair was examined by neutral comet assays (*P < 0.05). Tail moments were summarized from three independent experiments with 50 cells for a single time point per sample. The data are presented as mean ± SEM. **B** The cells lacking OGA are sensitive to IR treatment. Colony formation assays were performed in U2OS cells expressing full-length OGA, N-OGA, or C-OGA. The cells were treated with indicated doses of IR. Representative images of colonies in plates stained with Giemsa. The results were summarized from three independent experiments. The data are presented as mean ± SEM.

Since OGA is the solo enzyme that hydrolases O-GlcNAcylation following DNA damage, OGA may play a critical role for DNA damage repair. To investigate the function of the catalytic domain and HAT domain of OGA in DNA damage repair, we used siRNA to knockdown OGA and reconstituted the cells with full-length OGA, N-OGA, or C-OGA (Supplemental Fig. S3). We treated cells with 10 Gy of IR to induce DSBs. We measured repair kinetics of DSB using comet assays under neutral conditions. Loss of OGA significantly suppressed DSB repair. In addition, loss of either the catalytic domain or the HAT domain of OGA, the cells were hypersensitive to IR treatment, suggesting that both the catalytic domain and the HAT domain of OGA plays important roles in DNA damage repair (Fig. 2A). Further, we performed cell cycle analysis of each sample by flow cytometry. Without DNA damage, we did not observe any cell cycle change (Supplemental Fig. S5A, B). However, following IR treatment, cells were arrested in S/G2 phases due to the activation of S/G2 checkpoints. After lesion repairing, the cell cycle arrest will be released. However, due to the repair defects in OGA-deficient cells, we found prolonged cell cycle arrest at S/G2 phase in these cells (Supplemental Fig. S5A, B), suggesting that OGA-mediated DNA damage repair and loss of OGA impaired DNA repair. Moreover, we treated the cells with different doses of IR and performed colony formation assays to examine the cell viability. Consistently, cells lacking full-length OGA or the key functional domains were hypersensitive to DNA damaging (Fig. 2B). Collectively, these results demonstrate that OGA participates in DNA damage repair. In particular, both the HAT domain and the catalytic domain are involved in DNA damage repair.

### The HAT domain of OGA recognizes substrates

**Fig. 3:**
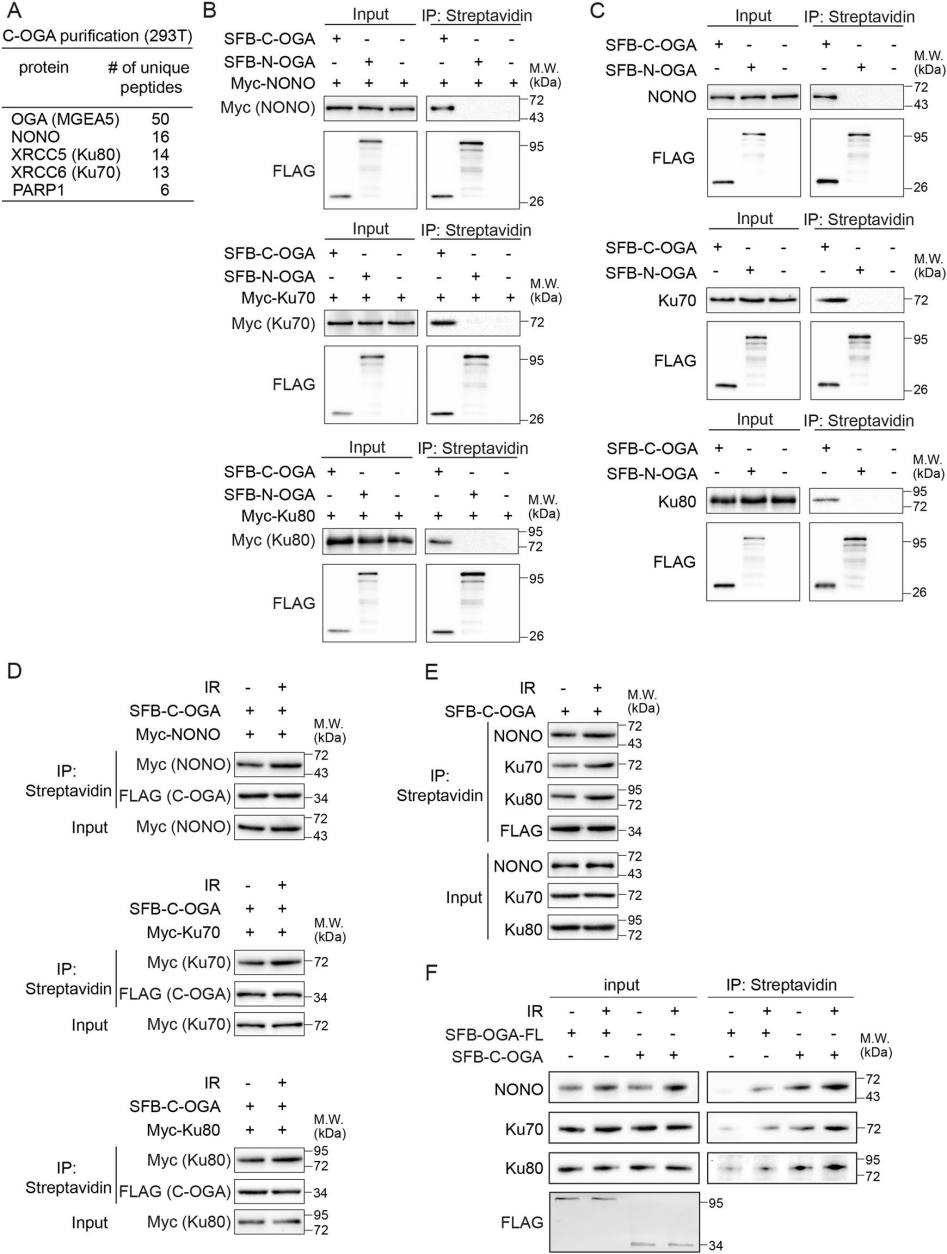
OGA associates with NONO and the Ku70/80 complex. **A** The list of proteins that were identified from the HAT domain of OGA (C-OGA) affinity purification and mass spectrometry. **B**, **C** The HAT domain of OGA (C-OGA) interacts with exogenous and endogenous NONO and the Ku70/80 complex. Myc-NONO and SFB-C-OGA or SFB-N-OGA were expressed in 293T cells. When endogenous NONO and the Ku70/80 complex were examined, only N-OGA or C-OGA was expressed in 293T cells. Cells were lyzed with NETN300 buffer. Cell extracts were examined by IP and western blot with indicated antibodies. **D**, **E** The interactions between the HAT domain of OGA (C-OGA) and NONO or the Ku70/80 complex were increased following IR treatment. Indicated constructs were expressed in 293T cells, and the cells were treated with 10 Gy of IR. The cell extracts were examined by IP and Western blot with indicated antibodies. SFB vector stands for SBP-FLAG-S tandem tag vector. **F** The interactions between the HAT domain of OGA (C-OGA) or OGA-FL and endogenous NONO or the Ku70/80 complex. C-OGA or OGA-FL was expressed in 293T cells, and the cells were treated with or without 10 Gy of IR. The cell extracts were examined by IP and Western blot with indicated antibodies.

To further dissect the function of the HAT domain of OGA, we searched for the binding partner(s) of the HAT domain using unbiased affinity purification and mass spectrometry analysis. We found that the HAT domain associated with several NHEJ repair factors such as NONO and the Ku70/80 complex from the unbiased protein affinity purification (Fig. 3A and Supplementary Table S1). Both NONO and the Ku70/80 complex play important roles in the NHEJ pathway for the DSB repair. However, the connection between these repair factors and O-GlcNAcylation or deglycosylation has not been examined. We examined the role of the interaction between the HAT domain and these NHEJ repair factors. Using the co-immunoprecipitation (co-IP) assays, we validated that the HAT domain of OGA (C-OGA) recognized these DNA damage repair factors but not N-OGA (Fig. 3B). We further examined the interaction between OGA and endogenous NONO or Ku70/80. Similarly, the HAT domain of OGA interacts these endogenous DNA damage repair factors but not N-OGA (Fig. 3C). Next, we asked if DNA damage regulated the interaction between the HAT domain of OGA and these DNA damage repair factors. We expressed Myc-NONO, Myc-Ku70, or Myc-Ku80 together with SFB-C-OGA. Following 10 Gy of IR treatment, we examined and found that the interactions between the HAT domain and these DNA damage repair factors were increased (Fig. 3D). To validate the results, we further examined the interaction between C-OGA and endogenous NONO or the Ku complex. Similarly, following IR treatment, the endogenous interactions were increased (Fig. 3E). We also examined and found that both of full-length OGA and HAT domain of OGA co-immunoprecipitated with NONO and Ku70/80 complex in response to IR (Fig. 3F).

**Fig. 4:**
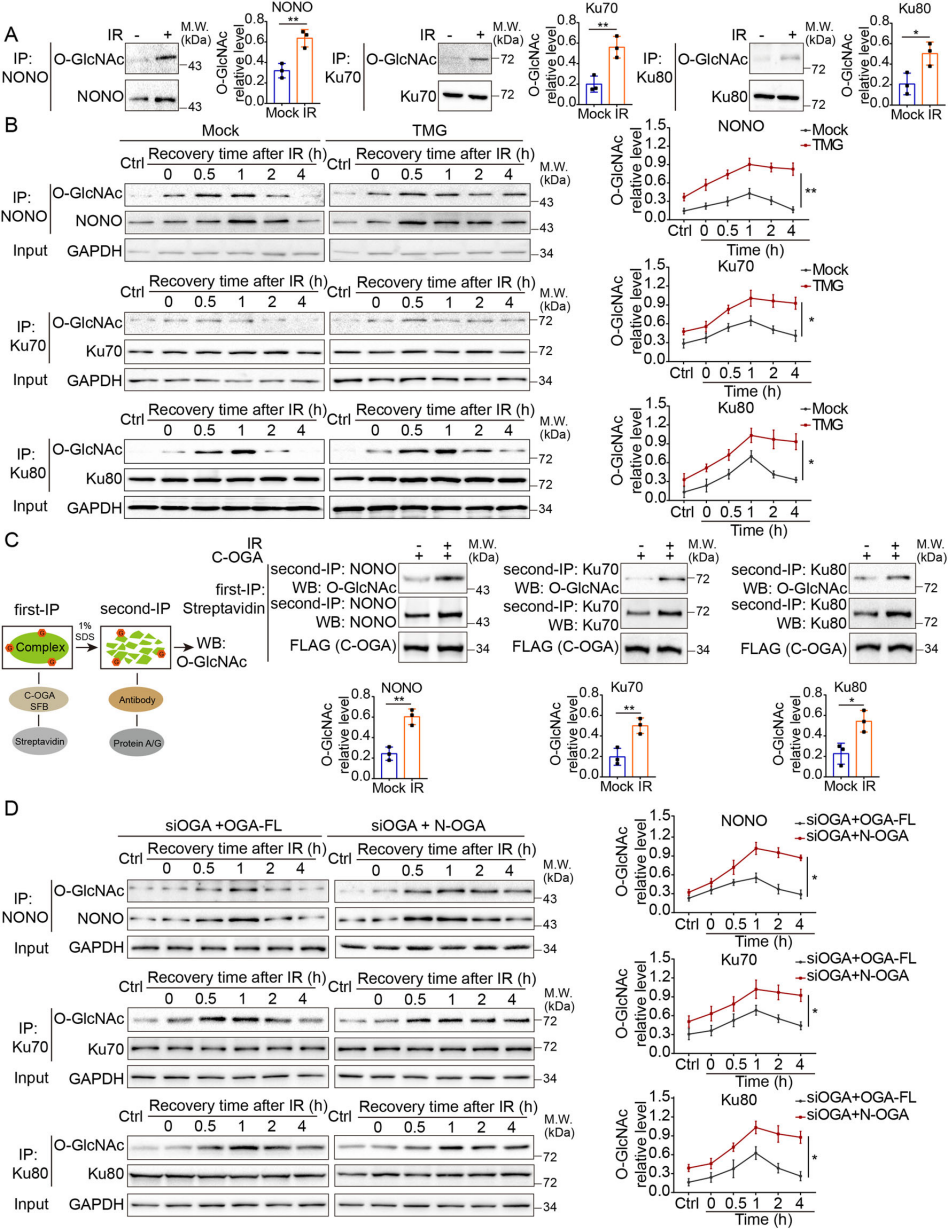
OGA mediates deglycosylation of NONO and the Ku70/80 complex. **A** The O-GlcNAcylation of NONO and the Ku70/80 complex were induced in response to DNA damage. 293T cells were treated with 10 Gy of IR and after 1 h cells were lyzed with NETN300 buffer. Cell extracts were examined by IP and western blot with indicated antibodies. The histogram shows the relative level of the O-GlcNAcylated NONO, Ku70, Ku80 signal. **B** Time course analyses of O-GlcNAcylation of NONO and the Ku70/80 complex events with mock or the TMG treatment following IR treatment using Western blotting. 293T cells were treated with mock or TMG and then exposed to 10 Gy of IR. Cell extracts were examined by IP and western blot with indicated antibodies. **C** The HAT domain of OGA recognizes O-GlcNAcylated NONO and the Ku70/80 complex following DNA damage. 293T cells were transfected with SFB-C-OGA and then two-step Co-IP assay was performed. Streptavidin beads were used to bind the HAT domain of OGA (C-OGA) partners in the first Co-IP assay. Anti-NONO, anti-Ku70/80 antibodies were used to IP target proteins in the second Co-IP assay. Western blot assay was performed with indicated antibodies. **D** Time course analyses of the deglycosylation of NONO and the Ku70/80 complex by full-length OGA or N-OGA following IR treatment using western blot. Cells were treated with siOGA and rescued with FL OGA or truncated OGA. Then these cells were exposed to 10 Gy of IR. Cell extracts were examined by IP and western blot with indicated antibodies.

Next, we treated 293T cells with 10 Gy of IR and examined the O-GlcNAcylation status of NONO and the Ku70/80 complex. Following IR treatment, both NONO and the Ku70/80 complex were O-GlcNAcylated (Fig. 4A). Time course analyses show that these O-GlcNAcylation events started immediately following IR treatment, reached to the peak levels at around one hour after DNA damage, and then gradually reduced. Moreover, with TMG treatment to suppress the enzymatic activity of OGA, these O-GlcNAcylation events were remarkably prolonged (Fig. 4B), suggesting that OGA mediates the deglycosylation of NONO and the Ku70/80 complex during DNA damage repair. Moreover, the O-GlcNAcylation level was remarkably increased in the HAT domain-associated species (Fig. 4C), suggesting that the HAT domain recognizes O-GlcNAcylated NONO and the Ku70/80 complex following DNA damage. In addition, we examined and found that the interactions between OGA and NHEJ factors are dependent on glycosylation (Supplemental Fig. S6).

Next, we examined the deglycosylation of NONO and the Ku70/80 complex. We used siRNA to knockdown OGA and reconstituted the cells with full-length OGA, N-OGA. Although the N-OGA still retains the enzymatic activity in vitro (Fig. 1C), we found that in the cells expressing the N-OGA, the deglycosylation of NONO and the Ku70/80 complex was disrupted during DNA damage repair (Fig. 4D). Collectively, these results suggest that the HAT domain of OGA might facilitate deglycosylation via the substrates recognition.

In addition, we examined the expression levels of the FL and truncated OGA before and after DNA damage and following OGA inhibitor treatments. We found that DNA damaging and OGA inhibitor treatments only slightly increased the expression of the FL and truncated OGA (Supplemental Fig. S7). In contrast, we examined and found that OGA knockdown slightly decreased OGT protein level (Supplemental Fig. S8). These minor expression changes may be due to stress responses under different conditions.

### Deglycosylation of NONO regulates its chromatin association and the NHEJ repair

**Fig. 5:**
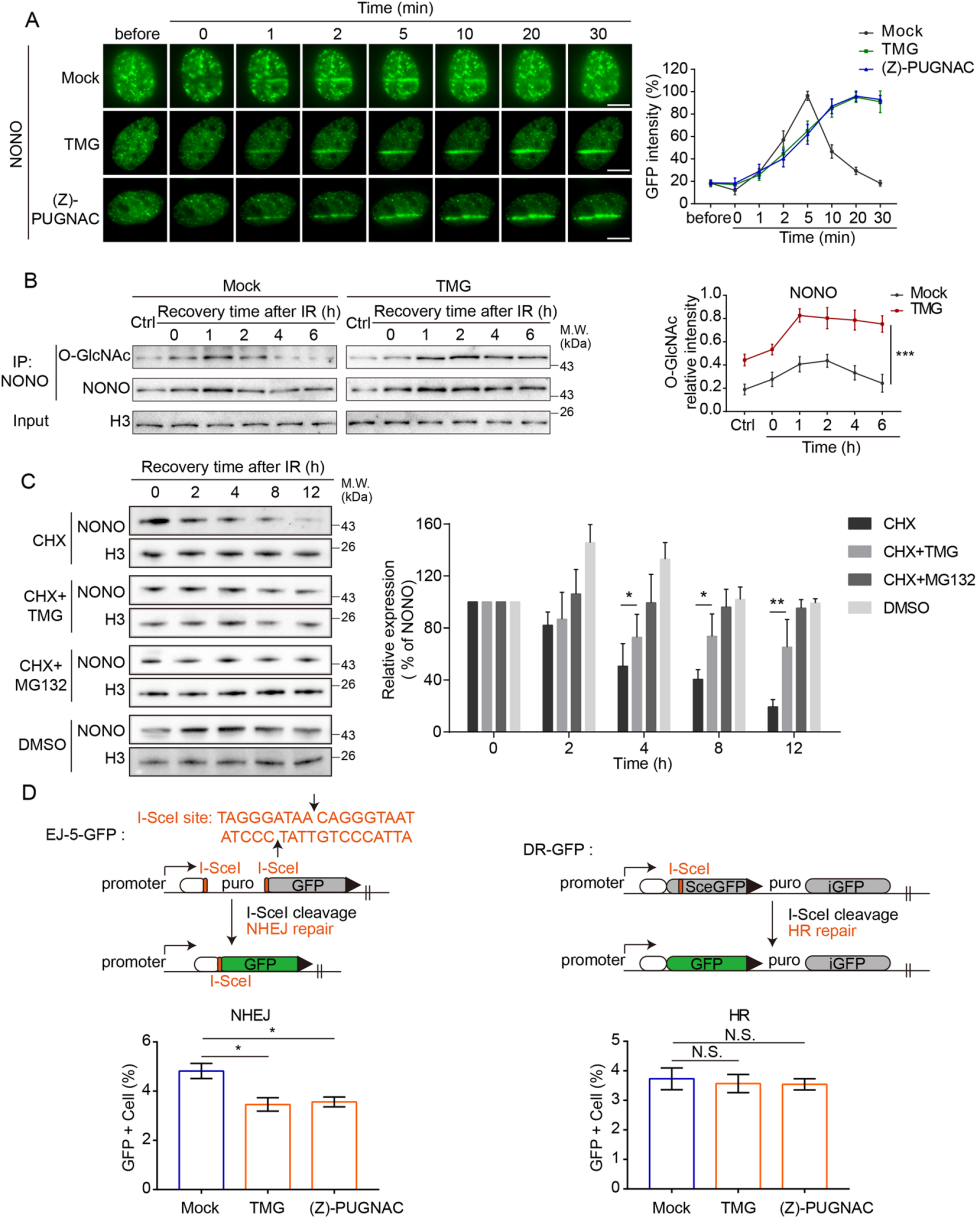
Deglycosylation of NONO regulates its chromatin association and NHEJ repair. **A** The OGA inhibitors’ treatment prolongs the retention of NONO at DNA lesions. GFP-NONO was expressed in U2OS cells, and the cells were treated with TMG or (Z)-PUGNAC followed by laser microirradiation. The relocation kinetics of NONO was examined in a time course. Scale bar: 5 μm. **B** TMG treatment prolongs the retention of NONO on chromatin during DNA damage response. 293T cells were treated with TMG followed by IR. The chromatin fractions were extracted. O-GlcNAcylation of NONO was examined in a time course with Western blotting. **C** TMG treatment suppresses the removal of NONO from the chromatin. 293T cells were treated with cycloheximide (CHX: 100 μM) or CHX and TMG (10 μM), and then collected at different time points. The chromatin fractions were examined with Western blotting. **D** The role of OGA in the NHEJ and HR repair pathway. GFP reporter assays were used to examine NHEJ and HR. Stable reporter cell lines were used to monitor either NHEJ or HR repair. Each of these cell lines has an integrated cassette comprising an I-SceI cleavage site, upon repair by either NHEJ or HR, restores GFP expression. Cells with mock or OGA inhibitors (TMG or (Z)-PUGNAC) treatment were assessed for each repair mechanism as indicated by the percentage of cells that express GFP.

Since NONO and the Ku70/80 complex participated in DSB repair, we further explored the biological functions of these deglycosylation events in the context of DSB repair. Following laser microirradiation, both NONO and the Ku70/80 complex quickly relocated to DNA lesions (Fig. 5A, Supplemental Fig. S9A, B). Although O-GlcNAcylation was not required for the recruitment of these DNA damage repair factors, we found that using TMG to suppress deglycosylation, the retention of NONO at DNA lesions was remarkably prolonged. The results were validated by the treatment of another OGA inhibitor (Z)-PUGNAC (Fig. 5A). We extracted chromatin fractions of mock cells or cells with TMG treatment and performed IP and western blot assay. Consistently, the retention of glycosylated NONO on the chromatin was prolonged as well (Fig. 5B). Different from NONO, O-GlcNAcylation status did not affect the Ku70/80 complex at DNA lesions because unlike NONO, the Ku70/80 complex stayed at DNA lesions for a prolonged time even without TMG treatment (Supplemental Fig. S9A, B).

To further examine the chromatin dissociation of NONO, we treated the cells with cycloheximide (CHX) to block protein synthesis. As an early DSB response factor, NONO was timely degraded in response to IR treatment (Fig. 5C), which is important to facilitate the subsequent loading of repair factors to repair DNA lesions in the next stage. Next, we included TMG and found that NONO degradation was suppressed (Fig. 5C), suggesting that blocking the removal of O-GlcNAcylation of NONO might impair the degradation of NONO.

Since NONO is involved in NHEJ repair [29, 30], we performed the GFP reporter assays and found that suppression of OGA-mediated deglycosylation by either TMG or (Z)-PUGNAC impaired NHEJ (Fig. 5D, Supplemental Fig. 10A). Consistent with prolonged DNA damage repair, we found that the cell cycle was arrested at S/G2 phase in cells loss of OGA activity (Supplemental Fig. 10B, C). To further confirm that OGA activity is required for NHEJ, we performed the rescue experiments in the GFP reporter assays (Supplemental Fig. 10D). In contrast, HR repair was not obviously impaired following these OGA inhibitor treatment. Taken together, these results suggest that suppression of OGA-mediated deglycosylation traps DNA damage repair factors such as NONO and impairs NHEJ repair.

## Discussion

OGA is the solo enzyme that hydrolases O-GlcNAcylation. In this study, we have demonstrated that OGA is enriched at the sites of DNA damage and participates in DNA damage repair. The recruitment of OGA to DNA lesions is dependent on its unique pseudo HAT domain. This HAT domain also recognizes the substrates of OGA and facilitates deglycosylation by capturing a set of substrates during DNA damage repair. To date, only the crystal structure of a truncated OGA comprising the catalytic domain and stalk domain has been solved [28]. Unfortunately, the structural information of the pseudo HAT domain of hOGA has not been available, and future structural analysis on the HAT will help in understanding the molecular mechanism underlying the substrate recognition and catalysis mediated by OGA.

**Fig. 6:**
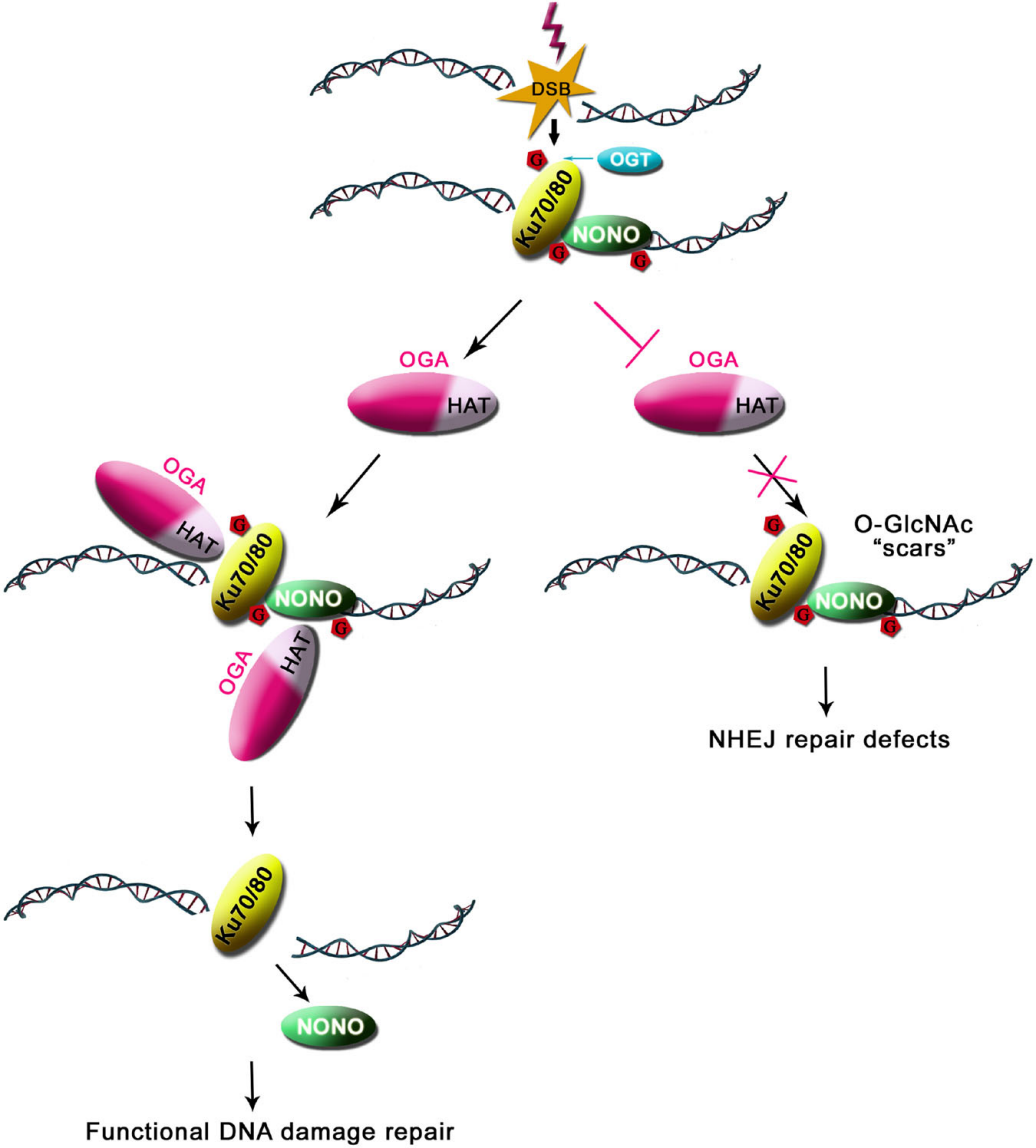
A working model of OGA-mediated deglycosylation in DNA damage repair. OGA recognizes O-GlcNAcylated substrates including NONO and the Ku complex via its pseudo HAT domain at DNA damage sites. OGA deglycosylates the substrates such as NONO and facilitates the timely removal of NONO for DSB repair.

In our study, we found that both NONO and the Ku70/80 complex are substrates of OGA and can be recognized by the HAT domain of OGA. These repair factors are known to initiate NHEJ [29, 31–35]. And suppression of OGA also impairs NHEJ, which is likely through the inhibition of deglycosylation of NONO and the Ku70/80 complex. We also found that suppression of OGA prolonged NONO retention at DNA damage sites and on the chromatin, which may delay NONO degradation and cause DSB repair defects. Due to the limitation of the mass spectrometry strategy, it is difficult to map all the O-GlcNAcylation sites on NONO and the Ku70/80 complex. Thus, the detailed mechanism by which the O-GlcNAcylation and deglycosylation events inducing the chromatin association and dissociation of NONO is still unclear. We speculate that the reversible O-GlcNAcylation induces the chromatin association and dissociation of NONO via two possible aspects: (1) Glycosylation modification changes the tertiary structure of NONO. Suppressed deglycosylation affects the higher-order tertiary structure of NONO, which results in impairing the dissociation of NONO from chromatin; (2) These O-GlcNAcylation events may crosstalk with other PTMs such as phosphorylation and ubiquitination during DNA damage repair, which may regulate the retention of NONO. A growing body of evidence demonstrates that O-GlcNAcylation regulates ubiquitination and protein stability [16]. In particular, it has been suggested that OGA is required for timely removing O-GlcNAcylation, which may facilitate DSB-induced ubiquitination [17, 36]. Thus, it is possible that O-GlcNAcylation controls the ubiquitination and stability of NONO. As a DNA repair factor in the early stage of repair, NONO is transiently recruited to DNA damage sites [31, 32]. It has been shown that NONO should timely disassociate from chromatin and play a protective role in DNA damage response [37]. We speculate that loss of de-O-GlcNAcylation regulation may leave some sugar “scars” on the early stage repair factors, which traps these factors at DNA lesions, thus in turn impairs normal DSB repair (Fig. 6). In addition, we could not exclude the possibility that O-GlcNAcylation of other chromatin modifiers indirectly regulates NONO in chromatin fraction. Nevertheless, OGA-dependent removal of O-GlcNAcylation may contribute for DSB repair and this dynamic and reversible modification is a key regulator in response to DNA damage.

Interestingly, NONO glycosylation peaks at one hour after IR treatment (Fig. 4B and D) while NONO is released from DNA damage sites 20 min following laser microirradiation (Fig. 5A). It is possible that NONO at DNA lesions may only represent a fraction of the total NONO protein. However, a large fraction of NONO is O-GlcNAcylated following DNA damage but may not localize at DNA lesions. It has been shown that NONO plays a key role in RNA metabolism [38]. It is possible that NONO is glycosylated following DNA damage but regulates RNA metabolism in another nuclear compartment.

Although the deglycosylation mediates the removal of NONO, similar deglycosylation processes do not affect the chromatin retention of the Ku70/80 complex. It is possible that the Ku complex may associate with other factors such as DNAPK or DSB ends to retain at DNA lesions for a prolonged time. Nevertheless, we have provided the first evidence that OGA-mediated deglycosylation regulates key repair factors at DNA lesions. We addressed the question that how OGA is involved in DNA damage. We reasoned that OGA bind some substrates, such as DSB rejoining factors Ku70/80 and NONO through its pseudo HAT domain and recruited to DNA damage sites. Collectively, our study suggests that OGA-mediated deglycosylation plays a critical role in the normal cellular response to DNA damage. The HAT domain plays a key role for the deglycosylation, and timely removal of O-GlcNAcylation contributes to DSB repair. Systematical mass spectrometry on DNA damage-induced O-GlcNAcylation may reveal additional molecular mechanisms in the regulation of the repair process.

In addition, loss of OGA did not abolish deglycosylation during DNA damage response. It is possible that O-GlcNAcylated substrates are quickly degraded during DNA damage response, which results in loss of O-GlcNAcylation. In fact, DNA damage induces massive protein ubiquitination [39, 40], and O-GlcNAcylation is often associated with ubiquitination [41]. Thus, it is possible that O-GlcNAcylated substrates are quickly degraded via ubiquitin-proteasome pathway.

## Materials and methods

### Plasmids, antibodies, siRNAs, and chemical reagents

The full-length cDNA of human OGA, HAT domain of human OGA (C-OGA), and lacking HAT domain of human OGA (N-OGA) were subcloned into pEGFP-N1, Pet-28a, and SFB vector. The catalytic-dead mutant (DD174-175AA) of OGA (OGA-ED) was generated using the QuikChange site-directed mutagenesis kit (Stratagene). The full-length cDNA of human NONO, Ku70, and Ku80 were subcloned into pEGFP-N1 and pCMV-Myc vector. The primers were listed below:

OGA-FL-S:5′-ATGGTGCAGAAGGAGAGTCAAG-3′,

OGA-FL-A:5′-TCACAGGCTCCGACCAAGTATAAC-3′,

OGA-HAT(C-OGA)-S:5′-CTCTTTTTTCAGCCACCTCCAC-3′,

OGA-HAT(C-OGA)-A:5′-TCACAGGCTCCGACCAAGTATAAC-3′,

OGA-△HAT(N-OGA)-S:5′-ATGGTGCAGAAGGAGAGTCAAG-3′,

OGA-△HAT(N-OGA)-A:5′-ATCATTTGCCCCATCAATTG -3′,

NONO-FL-S:5′-ATGCAGAGTAATAAAACTTTTAAC-3′,

NONO-FL-A:5′-TTAGTATCGGCGACGTTTG-3′,

Ku70-FL-S:5′-ATGTCAGGGTGGGAGTCATATT-3′,

Ku70-FL-A:5′-TCAGTCCTGGAAGTGCTTGG-3′,

Ku80-FL-S:5′-ATGGTGCGGTCGGGGAATAAG-3′,

Ku80-FL-A:5′-CTATATCATGTCCAATAAATCGTCC-3′.

Antibodies used in this study include the following: anti-O-GlcNAc (CTD 110.6, Cell Signaling Technology), anti-PAR (Trevigen), anti-GAPDH (Proteintech), anti-OGA antibody (Proteintech), anti-NONO (Proteintech), anti-Ku70 (Proteintech), anti-Ku80 (Proteintech), anti-FLAG (Sigma), anti-Myc (Cell Signaling Technology), anti-H3 (Proteintech). siRNA sequences were used to target human OGA (5′- GAAATCTATCAGTACCTAGGA−3′ or 5′-TGAATAAATAATTTCAATTTG-3′). siRNAs were transfected into cells using oligofectamine2000 (Invitrogen) according to the manufacturer’s instructions. The OGT inhibitor (OSMI-1), the OGA inhibitors (TMG or (Z)-PUGNAc), MG132 was purchased from APExBIO, and CHX was purchased from Cell Signaling Technology. For the treatment, a final concentration of 20 μM OSMI-1, 10 μM TMG, 50 μM (Z)-PUGNAc, 20 μM MG132, 100 μM CHX was added to the cell culture medium at the indicated timepoints.

### Cell lysis, immunoprecipitation (IP), and western blotting

The cells were harvested at the indicated time points after relevant treatment and washed twice with phosphate-buffered saline (PBS). The cell pellets were subsequently resuspended in the NETN lysis buffer (20 mM Tris-HCl, pH 8.0, 150 mM NaCl, 1 mM EDTA and 0.5 % NP-40) or SDS lysis buffer (20 mM Tris-HCl, pH 8.0, 150 mM NaCl, 2 mM EDTA, 1% Triton X-100, and 1% SDS). Thereafter, the insoluble fractions were digested by Benzonase to extract the chromatin fraction, then subjected to electrophoresis or immunoprecipitation followed by Western blot or dot blot analysis.

### Recombinant proteins and in vitro fluorometric assays

Recombinant His-tagged full-length OGA protein, a truncated product lacking pseudo HAT domain (N-OGA), and a truncated product lacking N-terminal enzymatic domain (C-OGA) were purified from *Escherichia coli* cells. The in vitro O-GlcNAcylation digestion assay was carried out as previously described [22]. Specifically, recombinant His-OGA protein or two mutants was incubated with 4-MU-GlcNAc(from Sigma) as substrates in 25 μl reaction buffer (50 mM NaH_2_PO_4_, 100 mM NaCl and 0.1% BSA, pH 6.5) for 30 min at 37 °C. The reaction was stopped by 150 μl Glycine (200 mM, pH10.75). The progress of the reaction was determined by measuring the extent of 4-methylumbelliferone liberated as determined by fluorescence measurements using a Varian CARY Eclipse Fluorescence Spectrophotometer 96-well plate system and comparison to a standard curve of 4-methylumbelliferone under identical buffer conditions. Excitation and emission wavelengths of 368 nM and 450 nM were used, respectively, with 5 mm slit openings. All the experiments were repeated three times in triplicate.

### Laser micro-irradiation and microscope image acquisition

For laser microirradiation, cells were grown on 35-mm glass-bottom dishes (MatTek Corporation). Laser microirradiation was performed on OLYMPUS IX71 inverted fluorescence microscope with a Micropoint Laser Illumination and Ablation System (Photonic Instruments). For time-lapse microscopic analysis, cells were first transfected with corresponding plasmids. Then, green fluorescent protein (GFP) positive cells were subjected to microirradiation. The GFP strips were recorded at indicated time points and then analysed with Image J software. For the time-course analysis of laser microirradiation, samples were subjected to continuous microirradiation along certain paths within the indicated time interval. All the images were acquired with cellSens standard (Version 1.3) software under OLYMPUS IX71 inverted fluorescence microscope equipped with a UPlanSApo 60×/1.35 oil immersion objective at room temperature. Identical contrast and brightness adjustments were used on images for all given experiments.

### The establishment of stable cell lines and affinity purification of OGA-HAT-containing complexes

To establish cell lines stably expressing epitope-tagged proteins, 293T cells were transfected with plasmids encoding SFB-OGA-HAT (SFB-C-OGA). Twenty-four hours after transfection, the cells were split at the 1000:1 ratio and cultured in the medium containing selection antibiotics for 3 weeks. The individual antibiotics-resistant colonies were isolated and screened by Western blotting for the expression of OGA-HAT (SFB-C-OGA) protein. 293T cells stably expressing SFB-OGA-HAT domain were lysed with NETN buffer on ice for 10 min. Crude lysates were cleared by centrifugation at 14,000 rpm at 4 °C for 10 min, and supernatants were incubated with streptavidin-conjugated beads (Thermo). The immunocomplexes were washed three times with NETN buffer and then bead-bound proteins were eluted with 500 μl NETN buffer containing 2 mg/ml biotin (Sigma). The eluted supernatant was incubated with S protein beads (Millipore). The beads were washed three times with NETN buffer and subjected to SDS-PAGE. The comassie staining was performed to visualize the protein bands. Specific bands were excised, digested and the peptides were analyzed by a mass spectrometer.

### NHEJ/HR in vivo reporter assays

DR-GFP-U2OS cells and EJ5-GFP-U2OS cells were treated with DMSO, TMG, or (Z)-PUGNAC, respectively, then were transfected with JS-20(SceI) or empty vector. After 48 h, 2 × 10^5^ cells were analyzed for GFP-positive cells by flow cytometry to demonstrate the repair efficiency of homologous recombination (HR) and NHEJ. In flow cytometry figures, the *y*-axis is side-scattered light (SSC) to determine the granularity of cells. The *x*-axis is GFP signal intensity of cells. GFP-positive populations were gated to distinguish a group of cells with positive signals. Uninduced cells were included as negative controls. The results represent the mean value of triplicated replications in each experiment.

### Neutral comet assays

Single-cell gel electrophoretic comet assays were performed under neutral conditions. Briefly, 24 h after electroporation of the indicated plasmids, or transfection with the indicated siRNA, 293T cells were treated with or without IR (10 Gy) and recovered in normal culture medium for the indicated time at 37 °C. Cells were collected and rinsed twice with ice-cold PBS; 2 × 10^4^ cells per milliliter were combined with 1% LMAgarose at 40 °C at the ratio of 1:3 (v/v) and immediately pipetted onto slides. For cellular lysis, the slides were immersed in the neutral lysis solution (2% sarkosyl, 0.5 M Na_2_EDTA, 0.5 mg/mL proteinase K at pH 8.0) overnight at 37 °C in the dark followed by washing in the rinse buffer (90 mM Tris buffer, 90 mM boric acid, 2 mM Na_2_EDTA at pH 8.5) for 30 min with two repeats. Next, the slides were subjected to electrophoresis at 20 V (0.6 V/cm) for 25 min and stained in 2.5 mg/mL propidium iodide for 20 min. All images were taken with a fluorescence microscope and analyzed by Comet Assay IV software.

### Colony formation assay

Cell viability of 293T cells transfected with the indicated siRNAs was assessed by colony-forming assays. Briefly, a total of 1000 cells per condition were plated into a six-well plate. Cells were then exposed to ionizing radiation (1, 2 or 4 Gy) using a γ-irradiator (Gammacell-40; MDS Nordion). After 7 to 10 days, colonies were fixed with methanol, stained using methylene blue in methanol, extensively washed with PBS and counted. The surviving cell fractions were calculated by comparing the numbers of colonies formed in the irradiated cultures with those in untreated control.

### Cell cycle analysis by propidium iodide staining

Cells treated with TMG or IR were harvested and washed in PBS, then were fixed in cold 70% ethanol at −20 °C for overnight. Cells were collected by centrifugation at 1000 rpm for 2 min. The pellet was suspended in 500 μl PI-solution and incubated for 30 min at 37 °C. All samples were analyzed by flow cytometry.

## Supplementary information

Supplementary information
