# Supplementary material for: OGA is associated with deglycosylation of NONO and the KU complex during DNA damage repair
Source: Cell Death Dis. 2021 Jun 16;12(7):622. doi: 10.1038/s41419-021-03910-6 (PMC8209095; doi:10.1038/s41419-021-03910-6)
Supplement: Supplementary file 1 — Supplementary information [file 41419_2021_3910_MOESM1_ESM.pdf]

Supplemental Figure Legend

Figure S1. DNA damage induces O-GlcNAcylation and OGA-dependent deglycosylation. (A) IR treatment induces O-GlcNAcylation. U2OS cells were exposed to 10 Gy of IR. The cell lysates were examined by dot-blotting with anti-O-GlcNAc antibody (CTD110.6, upper left panel) in a time course assay. The lysates were examined by anti-PAR antibody (middle left panel) and anti-GAPDH (lower left panel) as controls. The dot intensity was examined by Image J and was presented in a line graph (right panel). The results represent the mean value of triplicated replications in each experiment. (B) Suppression of OGA increases overall levels of O-GlcNAcylation. U2OS cells were treated with siOGA or TMG followed by IR. Cells were treated with TMG for 24 hours before IR. The cell lysates were subjected to dot blotting with CTD110.6 (left panels). The dot intensity was examined by Image J and was presented in a line graph (right panel). The results represent the mean value of triplicated replications in each experiment. (C) The relative kinetics of deglycosylation during DNA damage response in figure S1B. (D) The recruitment kinetics of OGA at DNA lesions. GFP-OGA was expressed in U2OS cells. The cells were treated with laser microirradiation. The representative images were shown (left panel). The peak fluorescence density of OGA at the laser strips was quantified at indicated time points by Image J (right panel). Scale bar: 5 μm.

Figure S2. DNA damage induces O-GlcNAcylation in MCF10A cells. (A) IR treatment induces O-GlcNAcylation. (B) Suppression of OGA increases overall levels of O-GlcNAcylation. (C) The relative kinetics of deglycosylation during DNA damage response in figure S2B.

Figure S3. Expression of full length or truncated OGA in 293T cells. Cells were treated with siOGA in 293T cells and reconstituted with full length OGA, C-OGA or N-OGA. Protein expressions were examined by Western blot with indicated antibodies. Because the antibody used in the experiment only recognized 1-350aa of OGA, the antibody could not detect the signal of C-OGA.

Figure S4. Endogenous OGA is recruited to DNA lesions. The cells were treated with laser microirradiation and immunostained with anti-OGA antibody.

Figure S5. The effect of OGA knockdown on cell cycle analysis. (A) Representative flow cytofluorometric plots of cell cycle analysis for the indicated cell groups. Cells were collected at the indicated time point, and cell cycle was analyzed by flow cytometry. One representative result of three independent experiments is shown. (B) Statistical analysis of cell cycle.

Figure S6. The interactions between OGA and NONO or Ku complex are dependent on glycosylation. Cells were treated with OGT inhibitor (OSMI-1) and the interactions between OGA and NONO or Ku70/80 were examined with IP and Western blot.

Figure S7. The expression level of OGA following DNA damaging treatment and OGA inhibitor treatment. Cells were treated with or without TMG and with or without 10 Gy of IR. Cell lysates were extracted and subjected to Western blot with indicated antibodies.

Figure S8. The expression of OGT in OGA knockdown cells.

Figure S9. OGA inhibitor treatment does not affect the recruitment of the Ku complex to DNA damage sites. GFP-Ku70 or GFP-Ku80 was expressed in U2OS cells. The cells were treated with TMG treatment (10 μM for 24h), followed by laser microirradiation. The recruitment kinetics of Ku70 (A) and Ku80 (B) were examined. The fluorescence density of Ku70 or Ku80 at the laser strips was quantified at indicated time points by Image J. Scale bar: 5 μm.

Figure S10. OGA regulates NHEJ. (A) Representative flow cytofluorometric plots

for figure 5D. (B) Representative flow cytofluorometric plots of cell cycle analysis for the indicated cell groups. Cells were treated with mock or TMG and then with 5Gy of IR treatment. The indicated cell groups were collected at the indicated time points, and analyzed by flow cytometry. (C) Statistical analysis of cell cycle of indicated samples in figure S10B. Cell cycle analysis of indicated samples were performed by flow cytometry. Three independent experiments were performed. (D) The role of OGA in the NHEJ repair pathway. GFP reporter assays were used to examine NHEJ. Cells were treated with siOGA and rescued with FL OGA or truncated OGA and then were assessed for NHEJ.

Table S1. Affinity purification and mass spectrometry analysis of OGA-HAT in 293T cells.

Figure S1

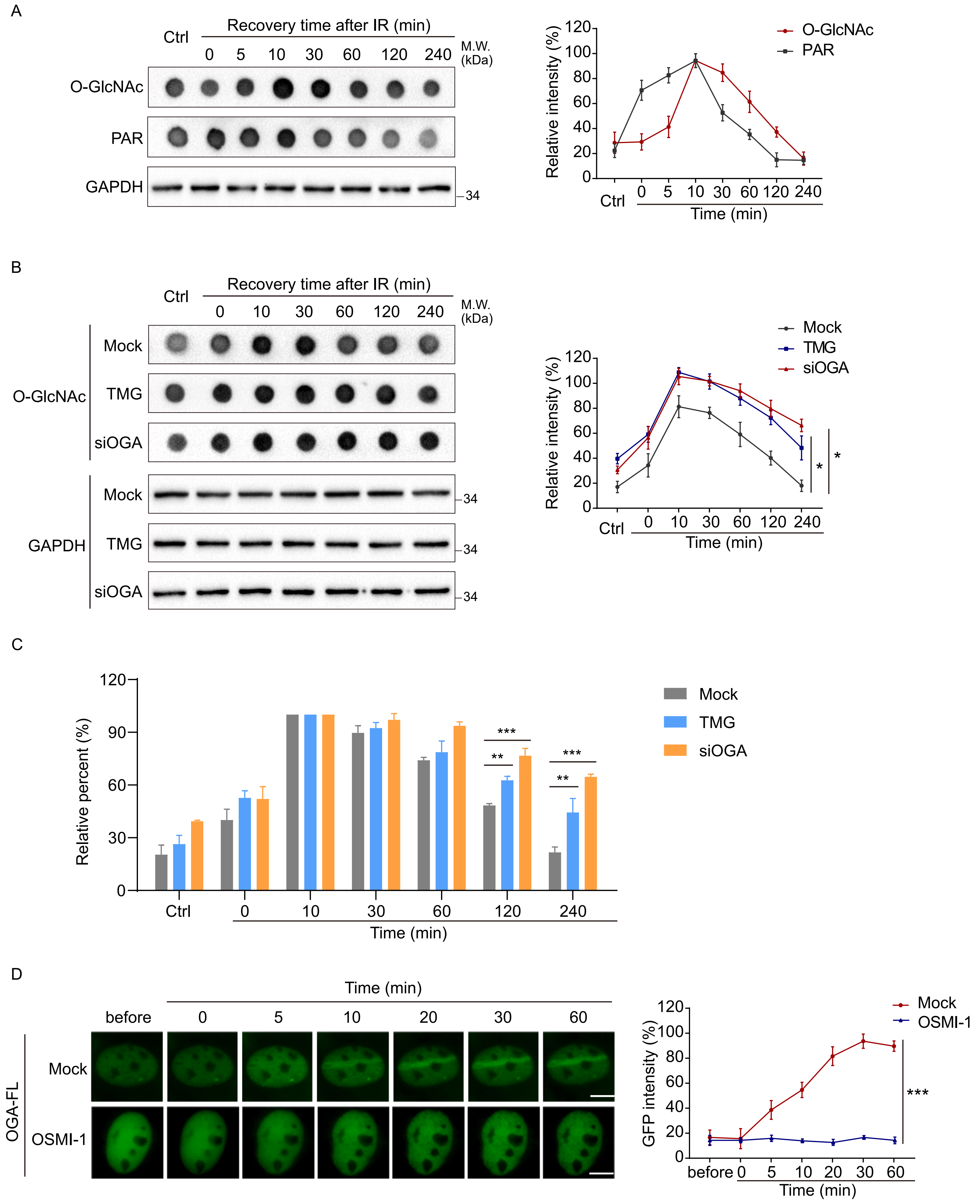
A
Ctrl
Recovery time after IR (min)
0 5 10 30 60 120 240
M.W. (kDa)
O-GlcNAc
PAR
GAPDH
34
Relative intensity (%)
120 100 80 60 40 20 0
O-GlcNAc
PAR
Ctrl 0 5 10 30 60 120 240
Time (min)
B
Ctrl
Recovery time after IR (min)
0 10 30 60 120 240
M.W. (kDa)
O-GlcNAc
Mock
TMG
siOGA
GAPDH
Mock
TMG
siOGA
34
34
34
Relative intensity (%)
120 100 80 60 40 20 0
Mock
TMG
siOGA
*
*
Ctrl 0 10 30 60 120 240
Time (min)
C
Relative percent (%)
120 90 60 30 0
Mock
TMG
siOGA
***
**
***
**
Ctrl 0 10 30 60 120 240
Time (min)
D
Time (min)
before 0 5 10 20 30 60
OGA-FL
Mock
OSMI-1
GFP intensity (%)
120 100 80 60 40 20 0
Mock
OSMI-1
***
before 0 5 10 20 30 60
Time (min)

Figure S2

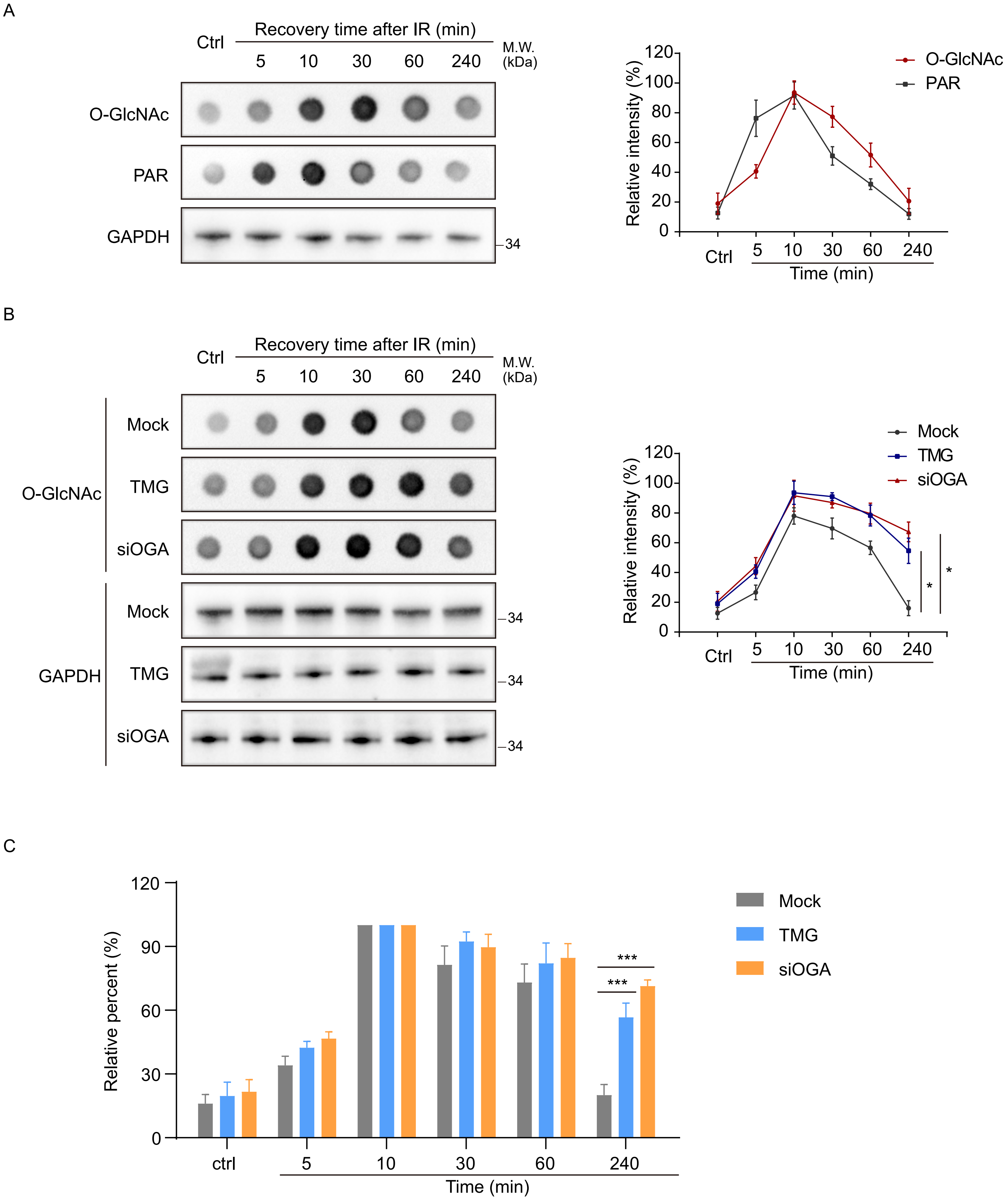
A
Ctrl
Recovery time after IR (min)
5
10
30
60
240
M.W.
(kDa)
O-GlcNAc
PAR
GAPDH
34
Relative intensity (%)
120
100
80
60
40
20
0
Ctrl
Time (min)
O-GlcNAc
PAR
B
Mock
TMG
siOGA
*
*
C
Relative percent (%)
90
30
ctrl
***
***

Figure S3

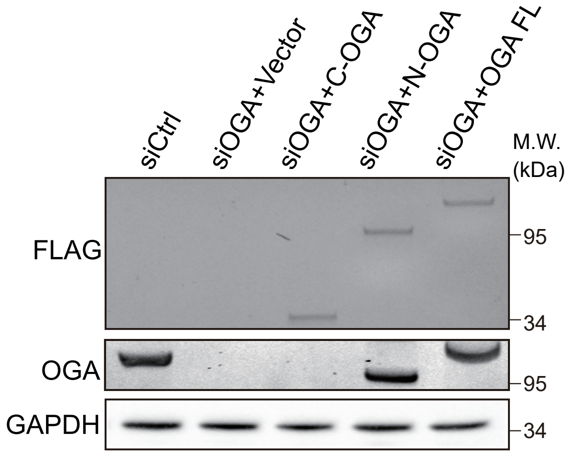
siCtrl
siOGA+Vector
siOGA+C-OGA
siOGA+N-OGA
siOGA+OGA FL
M.W.
(kDa)
FLAG
95
34
OGA
95
GAPDH
34

Figure S4

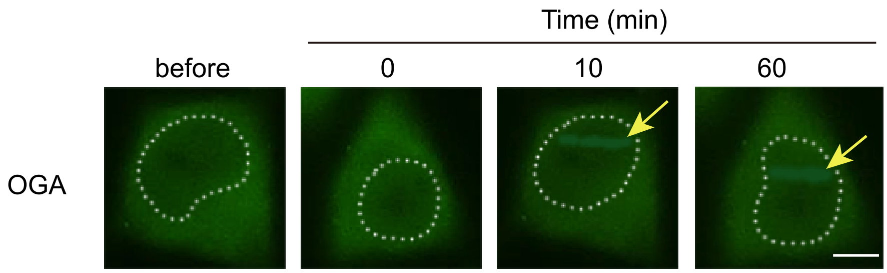

Figure S5

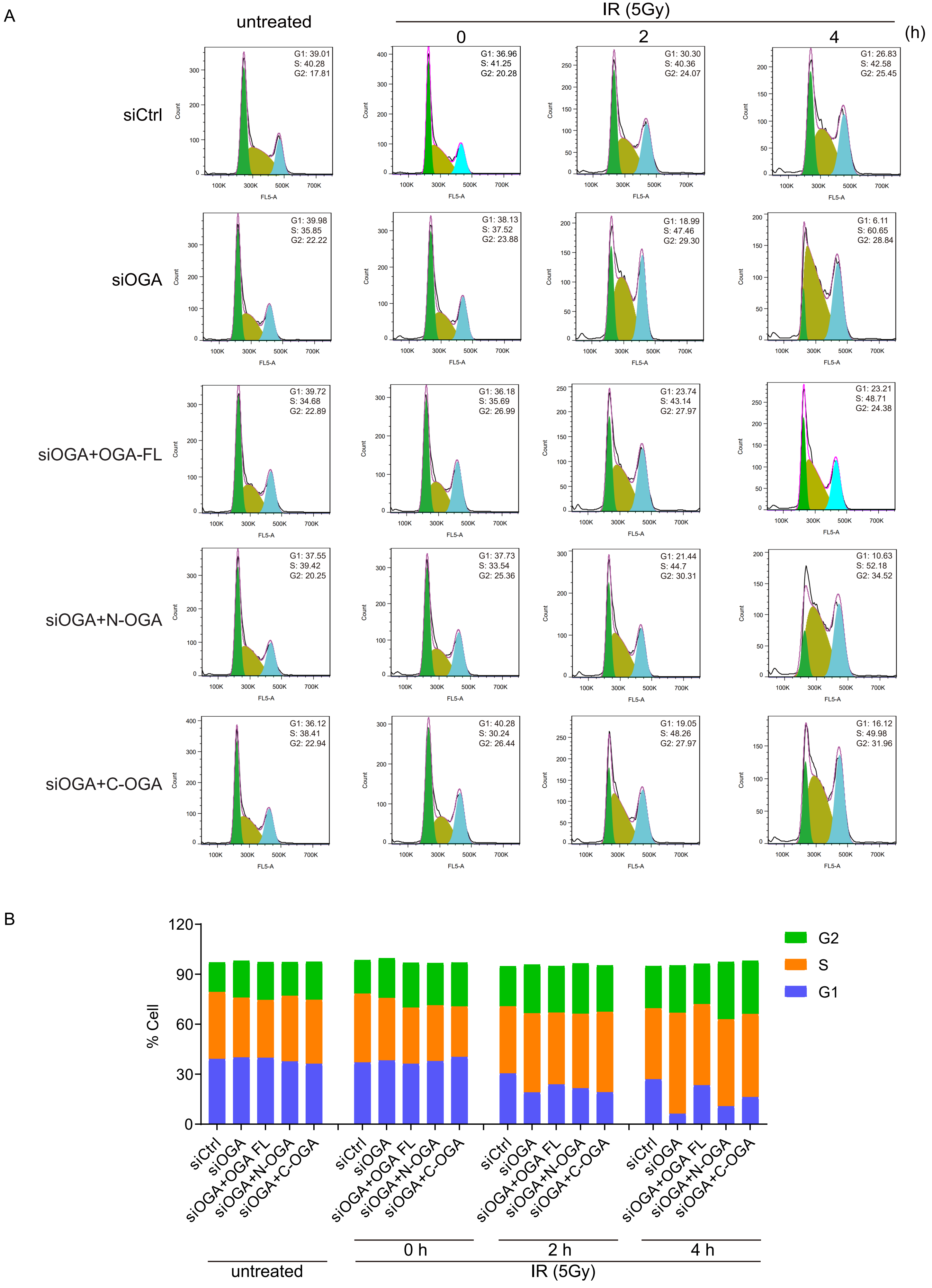
A
untreated
IR (5Gy)
0
2
4
(h)
siCtrl
siOGA
siOGA+OGA-FL
siOGA+N-OGA
siOGA+C-OGA
G1: 39.01
S: 40.28
G2: 17.81
G1: 36.96
S: 41.25
G2: 20.28
G1: 30.30
S: 40.36
G2: 24.07
G1: 26.83
S: 42.58
G2: 25.45
G1: 39.98
S: 35.85
G2: 22.22
G1: 38.13
S: 37.52
G2: 23.88
G1: 18.99
S: 47.46
G2: 29.30
G1: 6.11
S: 60.65
G2: 28.84
G1: 39.72
S: 34.68
G2: 22.89
G1: 36.18
S: 35.69
G2: 26.99
G1: 23.74
S: 43.14
G2: 27.97
G1: 23.21
S: 48.71
G2: 24.38
G1: 37.55
S: 39.42
G2: 20.25
G1: 37.73
S: 33.54
G2: 25.36
G1: 21.44
S: 44.7
G2: 30.31
G1: 10.63
S: 52.18
G2: 34.52
G1: 36.12
S: 38.41
G2: 22.94
G1: 40.28
S: 30.24
G2: 26.44
G1: 19.05
S: 48.26
G2: 27.97
G1: 16.12
S: 49.98
G2: 31.96
Count
FL5-A
B
% Cell
G2
S
G1
siCtrl
siOGA
siOGA+OGA FL
siOGA+N-OGA
siOGA+C-OGA
0 h
2 h
4 h
untreated
IR (5Gy)

Figure S6

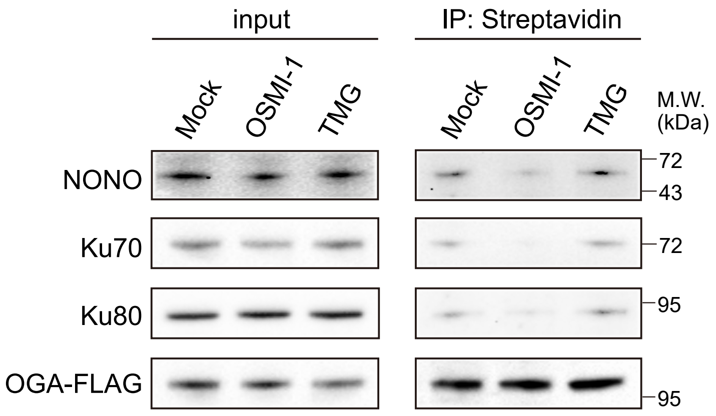
input
IP: Streptavidin
Mock
OSMI-1
TMG
Mock
OSMI-1
TMG
M.W. (kDa)
NONO
Ku70
Ku80
OGA-FLAG
72
43
72
95
95

Figure S7

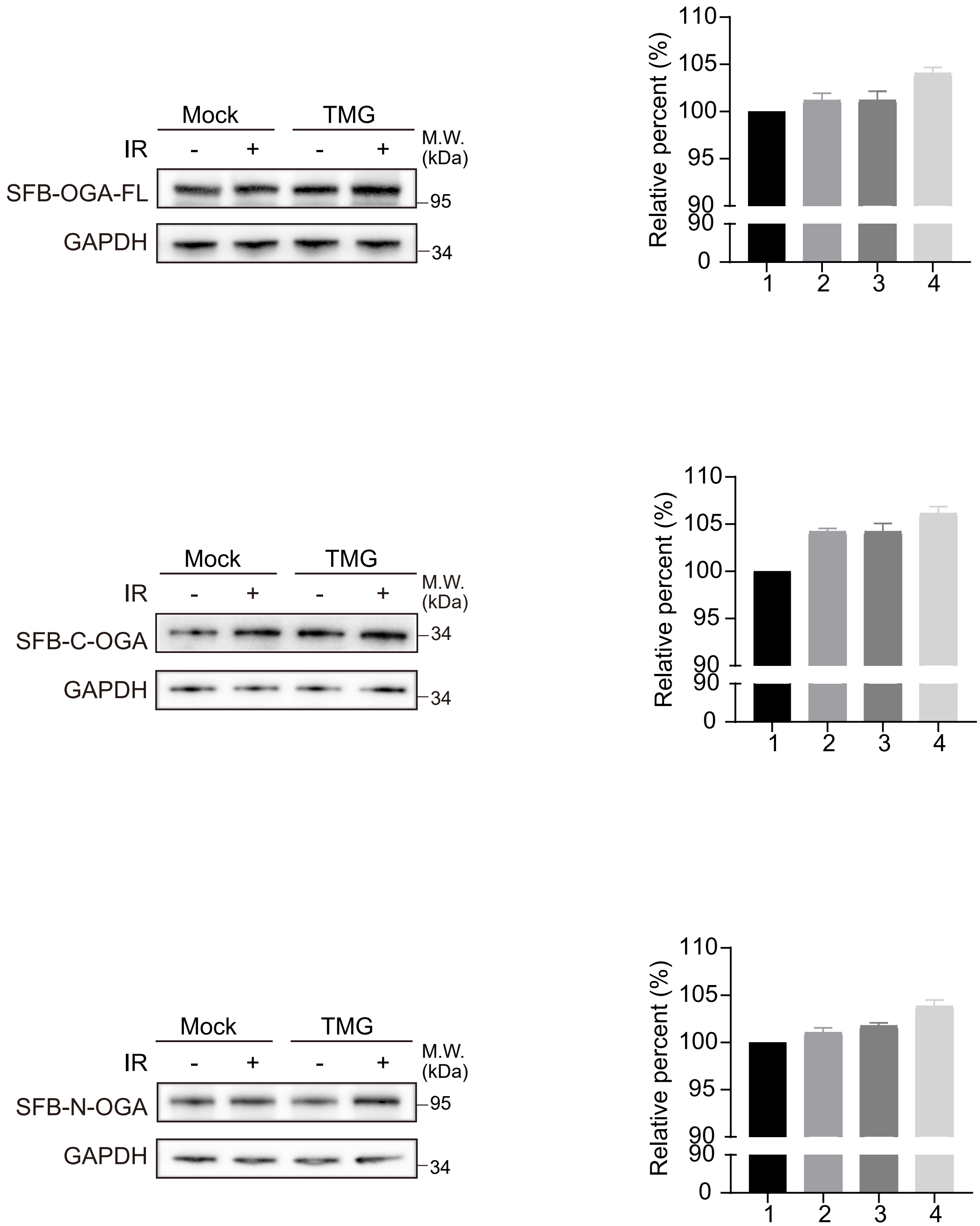

Mock
TMG
IR
-
+
-
+
M.W. (kDa)
SFB-OGA-FL
95
GAPDH
34
Relative percent (%)
110
105
100
95
90
90
0
1
2
3
4
Mock
TMG
IR
-
+
-
+
M.W. (kDa)
SFB-C-OGA
34
GAPDH
34
Relative percent (%)
110
105
100
95
90
90
0
1
2
3
4
Mock
TMG
IR
-
+
-
+
M.W. (kDa)
SFB-N-OGA
95
GAPDH
34
Relative percent (%)
110
105
100
95
90
90
0
1
2
3
4

Figure S8

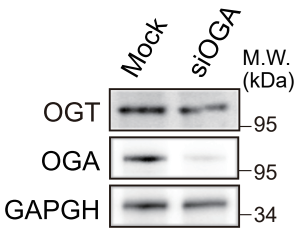

Figure S9

A

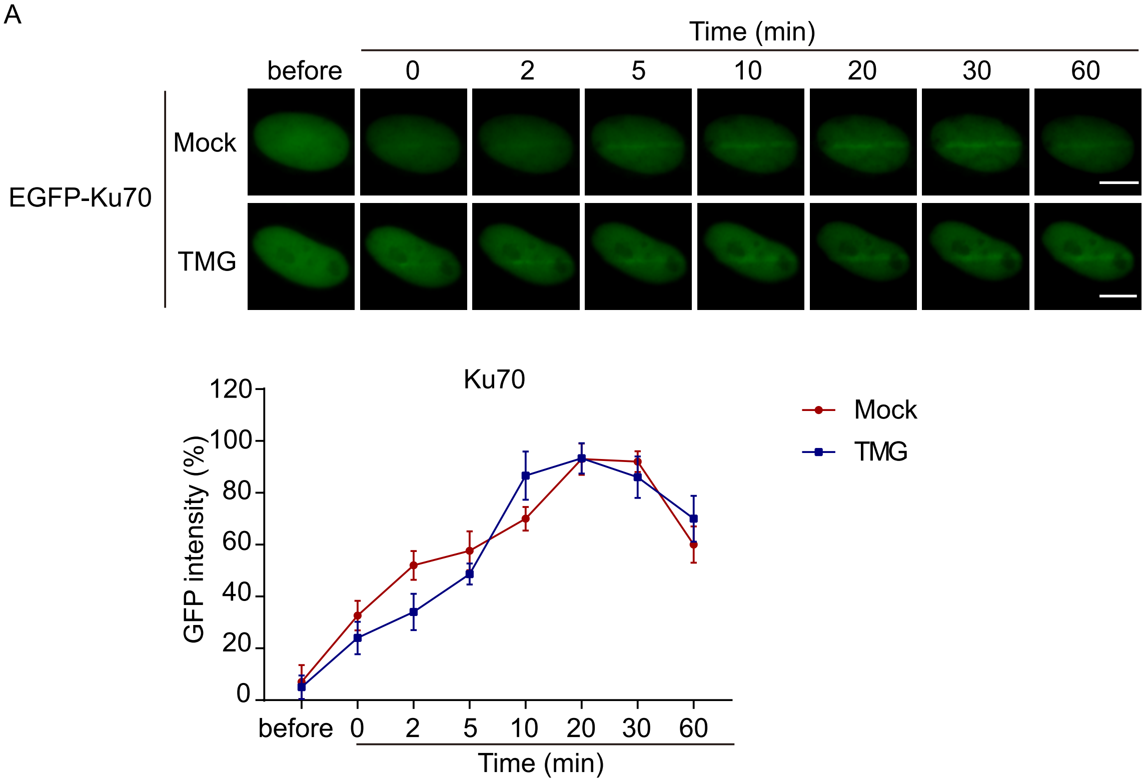

Time (min)
before
0
2
5
10
20
30
60
EGFP-Ku70
Mock
TMG
Ku70
GFP intensity (%)
120
100
80
60
40
20
0
Mock
TMG

B

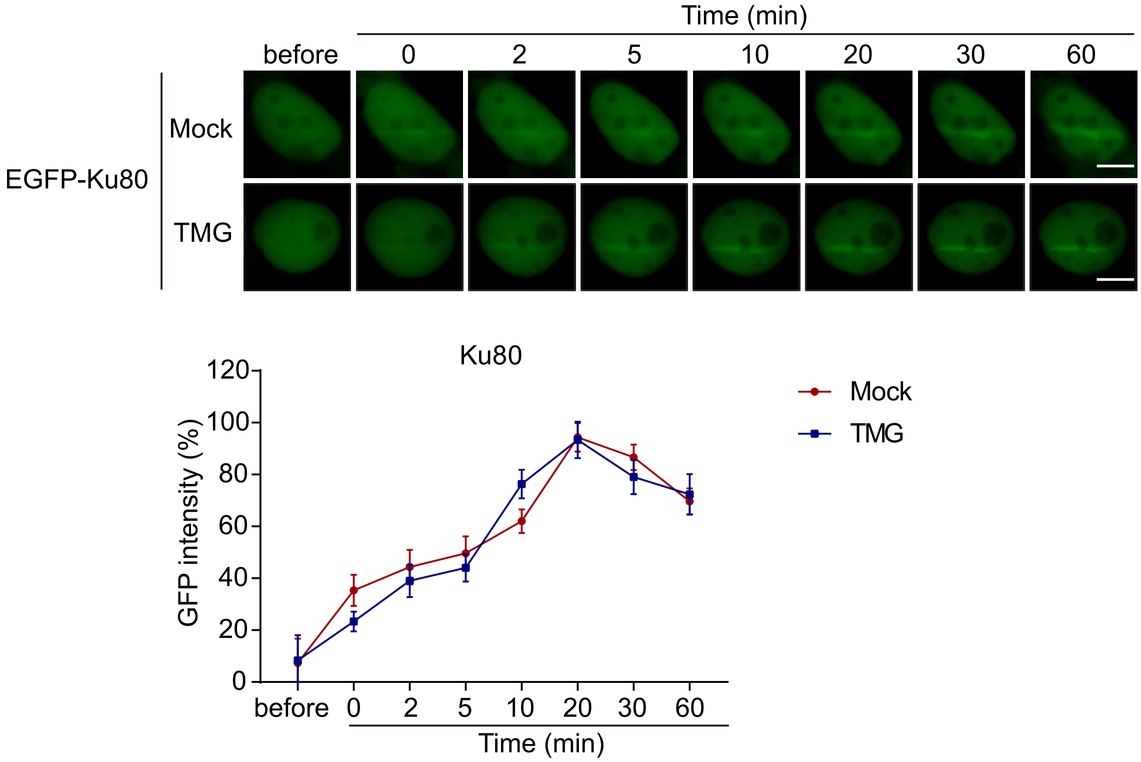

Time (min)
before
0
2
5
10
20
30
60
EGFP-Ku80
Mock
TMG
Ku80
GFP intensity (%)
120
100
80
60
40
20
0
Mock
TMG

Figure S10

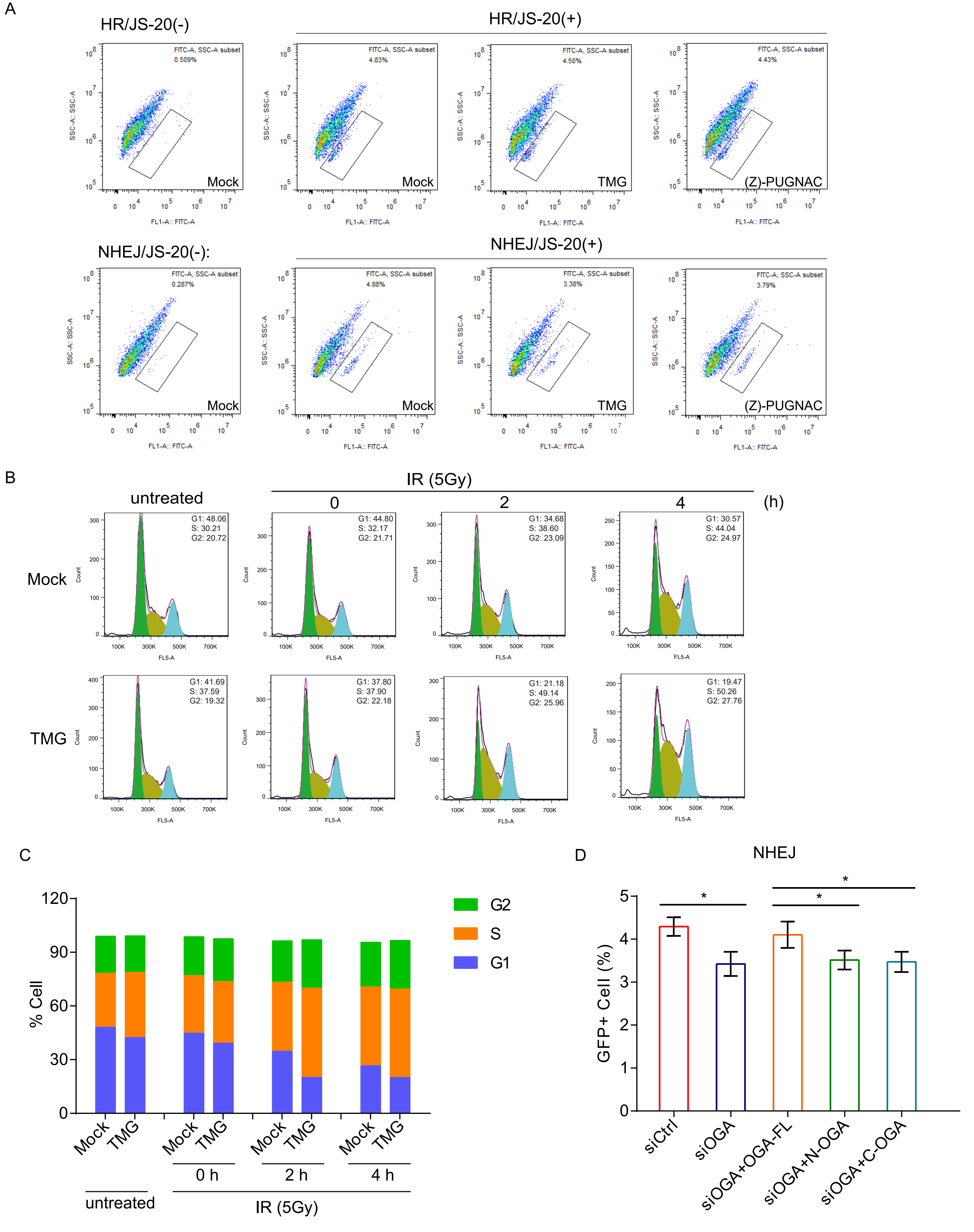
A
HR/JS-20(-)
HR/JS-20(+)
FITC-A, SSC-A subset
0.509%
4.83%
4.50%
4.43%
Mock
Mock
TMG
(Z)-PUGNAC
NHEJ/JS-20(-):
NHEJ/JS-20(+)
0.287%
4.88%
3.38%
3.79%
SSC-A:: SSC-A
FL1-A:: FITC-A
B
untreated
IR (5Gy)
0
2
4
(h)
Mock
TMG
G1: 48.06
S: 30.21
G2: 20.72
G1: 44.80
S: 32.17
G2: 21.71
G1: 34.68
S: 38.60
G2: 23.09
G1: 30.57
S: 44.04
G2: 24.97
G1: 41.69
S: 37.59
G2: 19.32
G1: 37.80
S: 37.90
G2: 22.18
G1: 21.18
S: 49.14
G2: 25.96
G1: 19.47
S: 50.26
G2: 27.76
Count
FL5-A
C
% Cell
G2
S
G1
Mock
TMG
0 h
2 h
4 h
untreated
IR (5Gy)
D
NHEJ
GFP+ Cell (%)
siCtrl
siOGA
siOGA+OGA-FL
siOGA+N-OGA
siOGA+C-OGA

Table S1

Unique	Total	reference	Gene Symbol	MWT(kDa)	AVG
50	417	sp|O60502|OGA_HUMAN	MGEA5	102.85	2.5627
29	114	sp|P11142|HSP7C_HUMAN	HSPA8	70.85	2.9457
29	75	sp|P68371|TBB4B_HUMAN	TUBB4B	49.8	3.0418
26	27	sp|Q9UJS0|CMC2_HUMAN	SLC25A13	74.13	3.1814
22	28	sp|P38646|GRP75_HUMAN	HSPA9	73.63	2.8294
22	25	sp|Q92598|HS105_HUMAN	HSPH1	96.8	3.0499
22	24	sp|P34932|HSP74_HUMAN	HSPA4	94.27	3.3323
22	22	sp|P42704|LPPRC_HUMAN	LRPPRC	157.81	2.7976
21	145	sp|P0DMV9|HS71B_HUMAN	HSPA1B	70.01	2.829
21	59	sp|Q9BQE3|TBA1C_HUMAN	TUBA1C	49.86	2.8113
21	24	sp|O95831|AIFM1_HUMAN	AIFM1	66.86	3.0061
20	20	sp|Q13085|ACACA_HUMAN	ACACA	265.38	2.8033
19	20	sp|P49411|EFTU_HUMAN	TUFM	49.51	2.7878
19	19	sp|O14980|XPO1_HUMAN	XPO1	123.31	3.0015
16	189	sp|P34931|HS71L_HUMAN	HSPA1L	70.33	2.6195
16	16	sp|P23396|RS3_HUMAN	RPS3	26.67	3.2879
16	21	sp|Q15233|NONO_HUMAN	NONO	54.2	2.9274
15	58	sp|P11021|GRP78_HUMAN	HSPA5	72.29	2.8999
15	15	sp|P06576|ATPB_HUMAN	ATP5B	56.52	3.1788
15	15	sp|O75746|CMC1_HUMAN	SLC25A12	74.71	3.1351
14	16	sp|P13010|XRCC5_HUMAN	XRCC5	82.65	2.2705
14	15	sp|O14654|IRS4_HUMAN	IRS4	133.68	2.9778
14	14	sp|P27708|PYR1_HUMAN	CAD	242.83	2.8259
13	15	sp|P05023|AT1A1_HUMAN	ATP1A1	112.82	3.1254
13	14	sp|P16615|AT2A2_HUMAN	ATP2A2	114.68	2.8503
13	14	sp|P12956|XRCC6_HUMAN	XRCC6	69.8	2.594
13	13	sp|P33993|MCM7_HUMAN	MCM7	81.26	2.9398
13	13	sp|Q9UNE7|CHIP_HUMAN	STUB1	34.83	2.8381
12	14	sp|Q00325|MPCP_HUMAN	SLC25A3	40.07	2.7054
12	13	sp|Q9NVI7|ATD3A_HUMAN	ATAD3A	71.32	2.8945
12	12	sp|P25705|ATPA_HUMAN	ATP5A1	59.71	3.5456
12	12	sp|Q02978|M2OM_HUMAN	SLC25A11	34.04	3.5031
12	12	sp|Q07065|CKAP4_HUMAN	CKAP4	65.98	3.1806
12	12	sp|O95573|ACSL3_HUMAN	ACSL3	80.37	3.0781
12	12	sp|Q92616|GCN1_HUMAN	GCN1	292.57	2.8608
11	17	sp|Q9UNF1|MAGD2_HUMAN	MAGED2	64.91	3.208
11	13	sp|P10809|CH60_HUMAN	HSPD1	61.02	3.47
11	11	sp|P11182|ODB2_HUMAN	DBT	53.45	2.9765
10	16	sp|P12236|ADT3_HUMAN	SLC25A6	32.85	2.8729
10	11	sp|P04843|RPN1_HUMAN	RPN1	68.53	2.9301
10	10	sp|O95757|HS74L_HUMAN	HSPA4L	94.45	3.5547
10	10	sp|O43175|SERA_HUMAN	PHGDH	56.61	3.4473
10	10	sp|P17987|TCPA_HUMAN	TCP1	60.31	2.7976
9	26	sp|P68104|EF1A1_HUMAN	EEF1A1	50.11	2.6006
9	11	sp|P53007|TXTP_HUMAN	SLC25A1	33.99	2.4305
9	10	sp|O00629|IMA3_HUMAN	KPNA4	57.85	3.3181
9	9	sp|O14983|AT2A1_HUMAN	ATP2A1	110.18	3.1076
9	9	sp|P17812|PYRG1_HUMAN	CTPS1	66.65	2.8934
9	9	sp|Q16531|DDB1_HUMAN	DDB1	126.89	2.4613
8	57	sp|P54652|HSP72_HUMAN	HSPA2	69.98	2.2407
8	10	sp|P31689|DNJA1_HUMAN	DNAJA1	44.84	3.6736
8	9	sp|Q9BVA1|TBB2B_HUMAN	TUBB2B	49.92	3.4124
8	8	sp|P08195|4F2_HUMAN	SLC3A2	67.95	3.3608

8	8	sp|Q8WVX9|FACR1_HUMAN	FAR1	59.32	3.262
8	8	sp|Q9Y230|RUVB2_HUMAN	RUVBL2	51.12	2.7649
8	8	sp|Q6P1M0|S27A4_HUMAN	SLC27A4	72.02	2.7646
7	9	sp|P62269|RS18_HUMAN	RPS18	17.71	2.4734
7	7	sp|Q9UL15|BAG5_HUMAN	BAG5	51.17	3.9543
7	7	sp|P00367|DHE3_HUMAN	GLUD1	61.36	2.5658
7	7	sp|P62249|RS16_HUMAN	RPS16	16.44	2.4838
7	7	sp|Q9Y4W6|AFG32_HUMAN	AFG3L2	88.53	2.3934
6	17	sp|P62736|ACTA_HUMAN	ACTA2	41.98	2.2518
6	9	sp|P53985|MOT1_HUMAN	SLC16A1	53.91	2.7097
6	7	sp|P13639|EF2_HUMAN	EEF2	95.28	3.1388
6	6	sp|Q9NVH1|DJC11_HUMAN	DNAJC11	63.24	3.6638
6	6	sp|P28288|ABCD3_HUMAN	ABCD3	75.43	3.4772
6	6	sp|P63261|ACTG_HUMAN	ACTG1	41.77	3.4147
6	6	sp|P35613|BASI_HUMAN	BSG	42.17	3.2728
6	6	sp|P19338|NUCL_HUMAN	NCL	76.57	3.2286
6	6	sp|P10155|RO60_HUMAN	TROVE2	60.63	3.177
6	6	sp|Q9Y285|SYFA_HUMAN	FARSA	57.53	3.1213
6	6	sp|Q96CS3|FAF2_HUMAN	FAF2	52.59	3.0877
6	6	sp|Q9Y265|RUVB1_HUMAN	RUVBL1	50.2	3.0787
6	6	sp|P49368|TCPG_HUMAN	CCT3	60.5	2.8951
6	6	sp|Q8IXB1|DJC10_HUMAN	DNAJC10	91.02	2.825
6	6	sp|P40939|ECHA_HUMAN	HADHA	82.95	2.7575
6	6	sp|P09874|PARP1_HUMAN	PARP1	113.01	2.7317
6	6	sp|Q53H12|AGK_HUMAN	AGK	47.11	2.6013
6	6	sp|P11172|UMPS_HUMAN	UMPS	52.19	2.5011
5	13	sp|P62987|RL40_HUMAN	UBA52	14.72	2.2503
5	12	sp|P07437|TBB5_HUMAN	TUBB	49.64	3.214
5	6	sp|Q58FF8|H90B2_HUMAN	HSP90AB2P	44.32	3.1193
5	5	sp|Q99615|DNJC7_HUMAN	DNAJC7	56.4	3.7723
5	5	sp|P49593|PPM1F_HUMAN	PPM1F	49.8	3.4819
5	5	sp|P08238|HS90B_HUMAN	HSP90AB1	83.21	3.1699
5	5	sp|Q9BUF5|TBB6_HUMAN	TUBB6	49.82	3.1004
5	5	sp|P52292|IMA1_HUMAN	KPNA2	57.83	3.0845
5	5	sp|Q8NF37|PCAT1_HUMAN	LPCAT1	59.11	2.7244
5	5	sp|P11586|C1TC_HUMAN	MTHFD1	101.5	2.6206
5	5	sp|O43592|XPOT_HUMAN	XPOT	109.89	2.5798
5	5	sp|Q9P035|HACD3_HUMAN	HACD3	43.13	2.5143
5	5	sp|P78371|TCPB_HUMAN	CCT2	57.45	2.4676
5	5	sp|Q9HCC0|MCCB_HUMAN	MCCC2	61.29	2.4444
5	5	sp|Q9Y3I0|RTCB_HUMAN	RTCB	55.17	2.4317
4	5	sp|Q16891|MIC60_HUMAN	IMMT	83.63	3.1159
4	4	sp|Q07021|C1QBP_HUMAN	C1QBP	31.34	3.8633
4	4	sp|Q96TA2|YMEL1_HUMAN	YME1L1	86.4	3.4762
4	4	sp|P26641|EF1G_HUMAN	EEF1G	50.09	3.2076
4	4	sp|Q9BSD7|NTPCR_HUMAN	NTPCR	20.7	3.0857
4	4	sp|Q9NZL4|HPBP1_HUMAN	HSPBP1	39.45	3.0715
4	4	sp|P07900|HS90A_HUMAN	HSP90AA1	84.61	3.0026
4	4	sp|Q9Y2L1|RRP44_HUMAN	DIS3	108.93	2.9808
4	4	sp|Q00839|HNRPU_HUMAN	HNRNPU	90.53	2.8733
4	4	sp|Q14974|IMB1_HUMAN	KPNB1	97.11	2.8712
4	4	sp|Q8IXI2|MIRO1_HUMAN	RHOT1	70.74	2.8651
4	4	sp|Q53GQ0|DHB12_HUMAN	HSD17B12	34.3	2.7526
4	4	sp|P15880|RS2_HUMAN	RPS2	31.3	2.7397

4	4	sp|Q9H936|GHC1_HUMAN	SLC25A22	34.45	2.7291
4	4	sp|Q9NXE4|NSMA3_HUMAN	SMPD4	93.29	2.7261
4	4	sp|P18085|ARF4_HUMAN	ARF4	20.5	2.5541
4	4	sp|Q96RQ3|MCCA_HUMAN	MCCC1	80.42	2.5374
4	4	sp|Q58FF7|H90B3_HUMAN	HSP90AB3P	68.28	2.5046
4	4	sp|Q8N2K0|ABD12_HUMAN	ABHD12	45.07	2.4823
4	4	sp|Q9UBX3|DIC_HUMAN	SLC25A10	31.26	2.4592
4	4	sp|Q9Y4R8|TELO2_HUMAN	TELO2	91.69	2.3905
3	5	sp|P62263|RS14_HUMAN	RPS14	16.26	2.9301
3	4	sp|Q15366|PCBP2_HUMAN	PCBP2	38.56	2.8448
3	3	sp|P04350|TBB4A_HUMAN	TUBB4A	49.55	3.9837
3	3	sp|P62829|RL23_HUMAN	RPL23	14.86	3.6344
3	3	sp|Q9NR30|DDX21_HUMAN	DDX21	87.29	3.4841
3	3	sp|Q13509|TBB3_HUMAN	TUBB3	50.4	3.3951
3	3	sp|Q58FF6|H90B4_HUMAN	HSP90AB4P	58.23	3.3943
3	3	sp|O60762|DPM1_HUMAN	DPM1	29.62	3.3771
3	3	sp|P50416|CPT1A_HUMAN	CPT1A	88.31	3.3654
3	3	sp|P11498|PYC_HUMAN	PC	129.55	3.3258
3	3	sp|Q3ZCQ8|TIM50_HUMAN	TIMM50	39.62	3.2794
3	3	sp|Q8TB36|GDAP1_HUMAN	GDAP1	41.32	3.0936
3	3	sp|P62753|RS6_HUMAN	RPS6	28.66	3.0747
3	3	sp|P62424|RL7A_HUMAN	RPL7A	29.98	3.0643
3	3	sp|Q9Y5M8|SRPRB_HUMAN	SRPRB	29.68	3.0123
3	3	sp|Q92621|NU205_HUMAN	NUP205	227.78	2.87
3	3	sp|P43307|SSRA_HUMAN	SSR1	32.22	2.84
3	3	sp|P62826|RAN_HUMAN	RAN	24.41	2.775
3	3	sp|P14678|RSMB_HUMAN	SNRPB	24.59	2.775
3	3	sp|P36542|ATPG_HUMAN	ATP5C1	32.98	2.7433
3	3	sp|Q9Y3Z3|SAMH1_HUMAN	SAMHD1	72.15	2.6637
3	3	sp|Q8IXI1|MIRO2_HUMAN	RHOT2	68.07	2.6277
3	3	sp|P40616|ARL1_HUMAN	ARL1	20.4	2.5309
3	3	sp|P10398|ARAF_HUMAN	ARAF	67.54	2.5008
3	3	sp|Q8N1F7|NUP93_HUMAN	NUP93	93.43	2.4923
3	3	sp|P05165|PCCA_HUMAN	PCCA	80.01	2.487
3	3	sp|P61619|S61A1_HUMAN	SEC61A1	52.23	2.4646
3	3	sp|P45880|VDAC2_HUMAN	VDAC2	31.55	2.4261
3	3	sp|P55209|NP1L1_HUMAN	NAP1L1	45.35	2.4159
3	3	sp|P52272|HNRPM_HUMAN	HNRNPM	77.46	2.2925
3	3	sp|Q92542|NICA_HUMAN	NCSTN	78.36	2.1456
2	4	sp|P05141|ADT2_HUMAN	SLC25A5	32.83	3.4334
2	3	sp|O60884|DNJA2_HUMAN	DNAJA2	45.72	3.2749
2	2	sp|Q7L8L6|FAKD5_HUMAN	FASTKD5	86.52	4.42
2	2	sp|Q9H2V7|SPNS1_HUMAN	SPNS1	56.59	4.0084
2	2	sp|Q14318|FKBP8_HUMAN	FKBP8	44.53	3.9026
2	2	sp|O15269|SPTC1_HUMAN	SPTLC1	52.71	3.7389
2	2	sp|P51571|SSRD_HUMAN	SSR4	18.99	3.5817
2	2	sp|P31040|SDHA_HUMAN	SDHA	72.65	3.3982
2	2	sp|Q9NPL8|TIDC1_HUMAN	TIMMDC1	32.16	3.3928
2	2	sp|O14929|HAT1_HUMAN	HAT1	49.48	3.3742
2	2	sp|Q9NZ01|TECR_HUMAN	TECR	36.01	3.2933
2	2	sp|Q9BT22|ALG1_HUMAN	ALG1	52.48	3.2501
2	2	sp|Q96JJ7|TMX3_HUMAN	TMX3	51.84	3.2283
2	2	sp|Q9Y5A9|YTHD2_HUMAN	YTHDF2	62.3	3.1747
2	2	sp|P62701|RS4X_HUMAN	RPS4X	29.58	3.1524

2	2	sp|Q14568|HS902_HUMAN	HSP90AA2P	39.34	3.0526
2	2	sp|O00505|IMA4_HUMAN	KPNA3	57.77	3.0038
2	2	sp|Q92499|DDX1_HUMAN	DDX1	82.38	2.9973
2	2	sp|Q9UG63|ABCF2_HUMAN	ABCF2	71.24	2.9405
2	2	sp|O95816|BAG2_HUMAN	BAG2	23.76	2.9229
2	2	sp|Q99832|TCPH_HUMAN	CCT7	59.33	2.8461
2	2	sp|P06733|ENOA_HUMAN	ENO1	47.14	2.8095
2	2	sp|P17980|PRS6A_HUMAN	PSMC3	49.17	2.7858
2	2	sp|P35998|PRS7_HUMAN	PSMC2	48.6	2.7727
2	2	sp|Q07020|RL18_HUMAN	RPL18	21.62	2.7374
2	2	sp|Q5T160|SYRM_HUMAN	RARS2	65.46	2.7253
2	2	sp|P42167|LAP2B_HUMAN	TMPO	50.64	2.6785
2	2	sp|P51784|UBP11_HUMAN	USP11	109.75	2.6632
2	2	sp|Q14683|SMC1A_HUMAN	SMC1A	143.14	2.6536
2	2	sp|P06748|NPM_HUMAN	NPM1	32.55	2.6307
2	2	sp|Q5VV42|CDKAL_HUMAN	CDKAL1	65.07	2.6296
2	2	sp|O00148|DX39A_HUMAN	DDX39A	49.1	2.6275
2	2	sp|Q99523|SORT_HUMAN	SORT1	92.01	2.6172
2	2	sp|P17844|DDX5_HUMAN	DDX5	69.1	2.5878
2	2	sp|Q96K37|S35E1_HUMAN	SLC35E1	44.74	2.5853
2	2	sp|Q643R3|LPCT4_HUMAN	LPCAT4	57.18	2.5782
2	2	sp|O75419|CDC45_HUMAN	CDC45	65.53	2.5768
2	2	sp|Q9UBM7|DHCR7_HUMAN	DHCR7	54.45	2.5535
2	2	sp|O00767|ACOD_HUMAN	SCD	41.5	2.444
2	2	sp|P55795|HNRH2_HUMAN	HNRNPH2	49.23	2.4302
2	2	sp|P83731|RL24_HUMAN	RPL24	17.77	2.1627
1	2	sp|P07858|CATB_HUMAN	CTSB	37.8	3.8663
1	1	sp|Q86VP6|CAND1_HUMAN	CAND1	136.29	4.5645
1	1	sp|O95299|NDUAA_HUMAN	NDUFA10	40.72	4.5369
1	1	sp|O60506|HNRPQ_HUMAN	SYNCRIP	69.56	4.2649
1	1	sp|P09622|DLDH_HUMAN	DLD	54.14	4.2576
1	1	sp|O00232|PSD12_HUMAN	PSMD12	52.87	4.2
1	1	sp|P55265|DSRAD_HUMAN	ADAR	135.98	4.1747
1	1	sp|Q9Y679|AUP1_HUMAN	AUP1	52.99	4.1443
1	1	sp|P61978|HNRPK_HUMAN	HNRNPK	50.94	4.1311
1	1	sp|Q6IAN0|DRS7B_HUMAN	DHRS7B	35.1	4.1107
1	1	sp|P08243|ASNS_HUMAN	ASNS	64.33	4.1018
1	1	tr|H7BXI1|H7BXI1_HUMAN	ESYT2	97.95	4.0007
1	1	sp|P52597|HNRPF_HUMAN	HNRNPF	45.64	3.9802
1	1	sp|P27635|RL10_HUMAN	RPL10	24.59	3.9582
1	1	sp|Q13748|TBA3C_HUMAN	TUBA3C	49.93	3.9121
1	1	sp|Q29836|1B67_HUMAN	HLA-B	40.32	3.8809
1	1	sp|P54709|AT1B3_HUMAN	ATP1B3	31.49	3.874
1	1	sp|P62913|RL11_HUMAN	RPL11	20.24	3.873
1	1	sp|P48651|PTSS1_HUMAN	PTDSS1	55.49	3.8598
1	1	sp|P08237|PFKAM_HUMAN	PFKM	85.13	3.8071
1	1	sp|Q12905|ILF2_HUMAN	ILF2	43.04	3.7859
1	1	sp|O15355|PPM1G_HUMAN	PPM1G	59.23	3.7653
1	1	sp|Q13885|TBB2A_HUMAN	TUBB2A	49.87	3.7617
1	1	sp|P57088|TMM33_HUMAN	TMEM33	27.96	3.7269
1	1	sp|P07384|CAN1_HUMAN	CAPN1	81.84	3.708
1	1	sp|A1L0T0|ILVBL_HUMAN	ILVBL	67.82	3.6885
1	1	sp|P38606|VATA_HUMAN	ATP6V1A	68.26	3.6807
1	1	sp|Q13724|MOGS_HUMAN	MOGS	91.86	3.6647

1	1	sp|P14625|ENPL_HUMAN	HSP90B1	92.41	3.6508
1	1	sp|P22695|QCR2_HUMAN	UQCRC2	48.41	3.6438
1	1	sp|Q99956|DUS9_HUMAN	DUSP9	41.84	3.6232
1	1	sp|Q9Y2X3|NOP58_HUMAN	NOP58	59.54	3.6162
1	1	sp|Q9UJZ1|STML2_HUMAN	STOML2	38.51	3.6065
1	1	tr|A0A024RBS1|A0A024RBS1_HUMAN	GCN1L1	266.71	3.6033
1	1	sp|Q14558|KPRA_HUMAN	PRPSAP1	39.37	3.5444
1	1	sp|Q02878|RL6_HUMAN	RPL6	32.71	3.5312
1	1	sp|P55060|XPO2_HUMAN	CSE1L	110.35	3.53
1	1	sp|P41250|GARS_HUMAN	GARS	83.11	3.523
1	1	sp|P04844|RPN2_HUMAN	RPN2	69.24	3.5149
1	1	sp|Q8NAN2|MIGA1_HUMAN	MIGA1	70.96	3.5046
1	1	sp|O43143|DHX15_HUMAN	DHX15	90.88	3.4648
1	1	sp|P16403|H12_HUMAN	HIST1H1C	21.35	3.4357
1	1	sp|P27816|MAP4_HUMAN	MAP4	120.93	3.415
1	1	sp|Q8WWC4|MAIP1_HUMAN	MAIP1	32.52	3.3793
1	1	sp|Q9Y5V3|MAGD1_HUMAN	MAGED1	86.11	3.3606
1	1	sp|Q16822|PCKGM_HUMAN	PCK2	70.68	3.3487
1	1	sp|P50990|TCPQ_HUMAN	CCT8	59.58	3.3364
1	1	sp|Q15054|DPOD3_HUMAN	POLD3	51.37	3.3198
1	1	sp|P56192|SYMC_HUMAN	MARS	101.05	3.2907
1	1	sp|Q9BVP2|GNL3_HUMAN	GNL3	61.95	3.2889
1	1	sp|P42677|RS27_HUMAN	RPS27	9.45	3.263
1	1	sp|Q9UJ14|GGT7_HUMAN	GGT7	70.42	3.2504
1	1	sp|Q9BRT8|CBWD1_HUMAN	CBWD1	44.04	3.2261
1	1	sp|P61962|DCAF7_HUMAN	DCAF7	38.9	3.2143
1	1	sp|Q9ULC5|ACSL5_HUMAN	ACSL5	75.94	3.1868
1	1	sp|Q96AG4|LRC59_HUMAN	LRRC59	34.91	3.1134
1	1	sp|P39656|OST48_HUMAN	DDOST	50.77	3.1097
1	1	sp|O14735|CDIPT_HUMAN	CDIPT	23.52	3.0963
1	1	sp|Q8NC54|KCT2_HUMAN	KCT2	29.22	3.0819
1	1	sp|Q86SK9|SCD5_HUMAN	SCD5	37.59	3.0376
1	1	sp|P63151|2ABA_HUMAN	PPP2R2A	51.66	3.0127
1	1	sp|P30837|AL1B1_HUMAN	ALDH1B1	57.17	2.9959
1	1	sp|P05166|PCCB_HUMAN	PCCB	58.18	2.9942
1	1	sp|Q96T76|MMS19_HUMAN	MMS19	113.22	2.9831
1	1	sp|Q6JQN1|ACD10_HUMAN	ACAD10	118.76	2.9386
1	1	sp|P23528|COF1_HUMAN	CFL1	18.49	2.9151
1	1	tr|E9PK54|E9PK54_HUMAN	HSPA8	19.94	2.9054
1	1	sp|P62258|1433E_HUMAN	YWHAE	29.16	2.9016
1	1	sp|Q68CQ7|GL8D1_HUMAN	GLT8D1	41.91	2.8945
1	1	sp|P62191|PRS4_HUMAN	PSMC1	49.15	2.8728
1	1	sp|O43390|HNRPR_HUMAN	HNRNPR	70.9	2.8726
1	1	sp|P46734|MP2K3_HUMAN	MAP2K3	39.29	2.8656
1	1	sp|Q01813|PFKAP_HUMAN	PFKP	85.54	2.8282
1	1	sp|O95373|IPO7_HUMAN	IPO7	119.44	2.8238
1	1	sp|Q05823|RN5A_HUMAN	RNASEL	83.48	2.8058
1	1	sp|Q6ZRP7|QSOX2_HUMAN	QSOX2	77.48	2.8021
1	1	sp|Q92841|DDX17_HUMAN	DDX17	80.22	2.7941
1	1	sp|P53618|COPB_HUMAN	COPB1	107.07	2.7928
1	1	sp|P11166|GTR1_HUMAN	SLC2A1	54.05	2.7916
1	1	sp|Q13148|TADBP_HUMAN	TARDBP	44.71	2.7735
1	1	sp|Q9NVE7|PANK4_HUMAN	PANK4	85.94	2.7606
1	1	sp|Q02086|SP2_HUMAN	SP2	64.86	2.7369

1	1	sp|P61204|ARF3_HUMAN	ARF3	20.59	2.7193
1	1	sp|Q9ULX6|AKP8L_HUMAN	AKAP8L	71.6	2.6966
1	1	sp|Q9H4I3|TRABD_HUMAN	TRABD	42.29	2.6914
1	1	sp|Q9H061|T126A_HUMAN	TMEM126A	21.51	2.6857
1	1	sp|P22102|PUR2_HUMAN	GART	107.7	2.673
1	1	sp|P13489|RINI_HUMAN	RNH1	49.94	2.6404
1	1	sp|P23634|AT2B4_HUMAN	ATP2B4	137.83	2.6265
1	1	sp|Q9UQE7|SMC3_HUMAN	SMC3	141.45	2.6112
1	1	sp|Q9Y6K0|CEPT1_HUMAN	CEPT1	46.52	2.611
1	1	sp|O75155|CAND2_HUMAN	CAND2	135.17	2.578
1	1	sp|O43542|XRCC3_HUMAN	XRCC3	37.83	2.5471
1	1	sp|Q9BRX2|PELO_HUMAN	PELO	43.33	2.5428
1	1	sp|Q16650|TBR1_HUMAN	TBR1	74.01	2.5419
1	1	sp|O00231|PSD11_HUMAN	PSMD11	47.43	2.5397
1	1	sp|O95429|BAG4_HUMAN	BAG4	49.56	2.5334
1	1	sp|Q14153|FA53B_HUMAN	FAM53B	45.74	2.5257
